# Evaluation of Risk Prediction with Hierarchical Data: Dependency Adjusted Confidence Intervals for the AUC

**DOI:** 10.3390/stats6020034

**Published:** 2023-04-24

**Authors:** Camden Bay, Robert J Glynn, Johanna M Seddon, Mei-Ling Ting Lee, Bernard Rosner

**Affiliations:** 1 Harvard Medical School, Brigham and Women’s Hospital, Boston, MA, 02115, USA; 2 University of Massachusetts Chan Medical School, Worcester, MA, 01655, USA; 3 University of Maryland School of Public Health, Department of Epidemiology and Biostatistics, College Park, MD, 20742, USA

**Keywords:** AUC, clustered data, confidence interval, correlated data, hierarchical models

## Abstract

The area under the true ROC curve (AUC) is routinely used to determine how strongly a given model discriminates between the levels of a binary outcome. Standard inference with the AUC requires that outcomes be independent of each other. To overcome this limitation, a method was developed for the estimation of the variance of the AUC in the setting of two-level hierarchical data using probit-transformed prediction scores generated from generalized estimating equation models, thereby allowing for the application of inferential methods. This manuscript presents an extension of this approach so that inference for the AUC may be performed in a three-level hierarchical data setting (e.g., eyes nested within persons and persons nested within families). A method that accounts for the effect of tied prediction scores on inference is also described. The performance of 95% confidence intervals around the AUC was assessed through the simulation of three-level clustered data in multiple settings, including ones with tied data and variable cluster sizes. Across all settings, the actual 95% confidence interval coverage varied from 0.943 to 0.958, and the ratio of the theoretical variance to the empirical variance of the AUC varied from 0.920 to 1.013. The results are better than those from existing methods. Two examples of applying the proposed methodology are presented.

## 1. Introduction

The area under the true receiver operating characteristic (ROC) curve, hereafter referred to as the AUC, is routinely estimated when it is of interest in determining how strongly a given model discriminates between the levels of a binary outcome. As described in Hanley and McNeil [3] and later modified by DeLong, DeLong, and Clarke-Pearson [1], the Mann–Whitney U statistic may be used to estimate the AUC and to compare the AUC between competing models. These approaches are only appropriate for inference with an AUC generated by data composed of independent observations. Extending the methodology of DeLong, DeLong, and Clarke-Pearson [1], Obuchowski [6] developed an approach for inference with the AUC that accounts for the clustering of data at two levels. In addition to this approach and building upon previous research that modified the Mann–Whitney U test for use with clustered data [7], Rosner, Qiu, and Lee [8] proposed a method for assessing the precision of and comparing AUC values in the setting of dependent data with a two-level structure (e.g., eyes measured within a person). In our application of the example discussed in Section 7, the AUC is the probability that a score for a random subunit with a negative outcome (i.e., a nonevent subunit) is lower than the score for a random subunit with a positive outcome (i.e., an event subunit).

To apply this approach, a transformation to normality of the model prediction scores is performed, so that a known null distribution and an alternative distribution distinguished by a location shift may be established. Then, by extending the Mann–Whitney U statistic to allow for data at two levels (e.g., eyes within a person), a closed-form expression of the variance of the AUC can be obtained.

**Figure 1.**
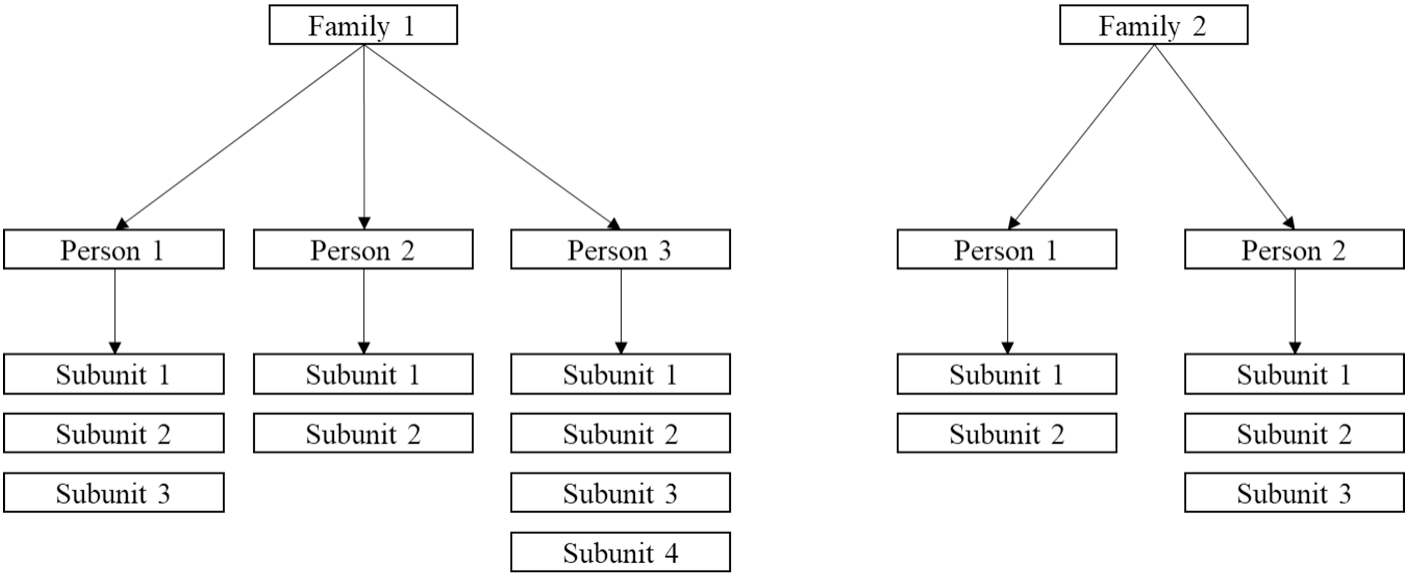
Example of a three-level hierarchy with 2 families and varying numbers of persons per family and subunits per person.

This article proposes further modification, enabling the construction of a confidence interval around the AUC with data having a three-level hierarchical structure. This structure can often be found in ophthalmology research. For example, when assessing the progression of age-related macular degeneration in datasets with multiple subjects in the same family, eyes are nested within patients, and patients may be nested within families [10]. As another example, if an investigator is using a Humphrey visual field analyzer to assess visual field loss, each patient will have data at multiple sites within each eye; this allows for the analysis of data at three levels (i.e., patient, eye, and site) [12]. In addition to ophthalmology, three-level data may be found in the fields of dentistry, where outcomes, such as the presence of caries, can occur within quadrants of the mouth, where each quadrant has multiple teeth, and in audiology, where ear-specific hearing loss may be studied in patients’ ears using tests with different frequencies within the same ear. In the following, the extended Mann–Whitney U statistic for three-level hierarchical data is discussed, the expectation and variance of this statistic, along with the construction of confidence intervals and the handling of ties, are defined, and simulation studies and two illustrations of its use are presented. For clarity, the levels of measurement will be referred to as family, person, and subunit. This terminology and hierarchy are illustrated in Figure 1.

## 2. Extended Mann-Whitney U Statistic for Three-Level Hierarchical Data

We consider a generalized linear mixed model having a Bernoulli-distributed response defined at the subunit level of the hierarchy, a logit link, and two nested random intercepts.

(1) $$ln\left(\frac{p_{ijk}|u_{i},v_{ij}}{1-p_{ijk}|u_{i},v_{ij}}\right)=\beta_{0}+\beta_{1}x_{ijk,1}+\beta_{2}x_{ijk,2}+\ldots +\beta_{m}x_{ijk,m}+u_{i}+v_{ij}$$

$$u_{i}∼N(0,\sigma_{u}^{2}),v_{ij}∼N(0,\sigma_{v}^{2})$$

where i, j, and k index the family, person, and subunit levels of the data, respectively, $p_{ijk}$ is the probability of a subunit experiencing an event, $\beta_{1}\ldots \beta_{m}$ are fixed effects and can be defined at any level of the hierarchy; $u_{i}$ is a family-level random effect; and $v_{ij}$ is a person-level random effect. The random effects are assumed to be independent of each other and independent of the fixed effects. Cluster sizes are allowed to vary at any level. Standard manipulations from the hierarchical modeling literature, such as decomposing fixed effects into within- and between-cluster effects and interactions, may be applied, but allowing for heteroscedasticity of the random effects’ variances or including random slopes is not viable, as the correlation structure must be strictly exchangeable. By fitting the above model defined in Equation (1) to the observed data, one can derive a composite score.


(2) $$S_{ijk}=\beta_{0}+\beta_{1}x_{ijk,1}+\beta_{2}x_{ijk,2}\ldots +\beta_{m}x_{ijk,m}$$

Let $\delta_{\text{ijk}}=1$ if the (i,j,k) subunit experiences an event 0 otherwise, and let I(a) be an indicator = 1 if $S_{i_{1}j_{1}k_{1}}<S_{i_{2}j_{2}k_{2}}$ is true and 0 otherwise. We define the AUC (or $\theta_{3}$) by:

(3) $$\theta_{3}=E[(1-\delta_{i_{1}j_{1}k_{1}})\delta_{i_{2}j_{2}k_{2}}I(S_{i_{1}j_{1}k_{1}}-S_{i_{2}j_{2}k_{2}})]$$

or in words, the probability that a risk score from a random subunit that has an event (or, in brief, an event subunit) is higher than a random nonevent subunit, which can be shown to be the area under a receiver operating characteristic curve (ROC), or AUC in brief. With 3-level hierarchical data, $\theta_{3}$, which is the parameter of interest, can be estimated with Equation (4):

(4) $$\hat{\theta_{3}}=\frac{\sum_{i_{1}=1}^{I}\sum_{j_{1}=1}^{J_{i_{1}}}\sum_{k_{1}=1}^{K_{i_{1}j_{1}}}\sum_{i_{2}=1}^{I}\sum_{j_{2}=1}^{J_{i_{2}}}\sum_{k_{2}=1}^{K_{i_{2}j_{2}}}(1-\delta_{i_{1}j_{1}k_{1}})\delta_{i_{2}j_{2}k_{2}}I(S_{i_{1}j_{1}k_{1}}-S_{i_{2}j_{2}k_{2}})}{\sum_{i_{1}=1}^{I}\sum_{j_{1}=1}^{J_{i_{1}}}\sum_{k_{1}=1}^{K_{i_{1}j_{1}}}\sum_{i_{2}=1}^{I}\sum_{j_{2}=1}^{J_{i_{2}}}\sum_{k_{2}=1}^{K_{i_{2}j_{2}}}(1-\delta_{i_{1}j_{1}k_{1}})\delta_{i_{2}j_{2}k_{2}}},i_{1}j_{1}k_{1}\neq i_{2}j_{2}k_{2}$$

where I is an indicator function that is equal to 1 if $S_{i_{1}j_{1}k_{1}}<S_{i_{2}j_{2}k_{2}}$ and 0 otherwise and δ is defined as the observed response status (1 = event, 0 = nonevent). Note that the numerator of Equation (4) is the generalization of the Mann–Whitney U statistics for observations across three levels of hierarchical data, e.g., across different families, across different subjects within the same family, and across different eyes of the same subjects. As usual, it is assumed that an increase in the prediction score is associated with an increase in the probability of having the event. The ties between prediction scores cannot be directly accommodated with this expression, however, they can be accounted for using the tie-breaking approach discussed in Section 4. Since the denominator of Equation (4) is equal to the total number of discordant pairs of subunit observations (i.e., one subunit exhibits an event and the other does not), $\hat{\theta_{3}}$ is defined as the proportion of discordant pairs where the observation with the event has a higher prediction score than the observation without. The observations belonging to the discordant pairs are allowed to be from different families or persons or within the same family or person.

To facilitate inference with $\hat{\theta_{3}}$, the prediction scores are transformed to a standard normal distribution. To do this, the probit (inverse of the cumulative standard normal probability distribution; $\Phi^{-1}$) is applied to the cumulative distribution of prediction scores (F), which may be estimated through the empirical distribution function. Note that this transformation preserves the rank of the prediction scores. The transformed prediction scores are defined as:

$$Z_{ijk}=\Phi^{-1}[F(S_{ijk})]$$

The transformed scores are distributed as:

Zijk∼{N(0,1)forδijk=0N(μ,1),μ≠0forδijk=1}

The 2 × 1 random vector of the differences between any two transformed prediction scores is as follows:

$$\left(\begin{array}{c} Z_{i_{1}j_{1}k_{1}}-Z_{i_{2}j_{2}k_{2}}\\ Z_{i_{3}j_{3}k_{3}}-Z_{i_{4}j_{4}k_{4}} \end{array}\right),Z_{i_{1}j_{1}k_{1}}\neq Z_{i_{2}j_{2}k_{2}}\;\text{and}\;Z_{i_{3}j_{3}k_{3}}\neq Z_{i_{4}j_{4}k_{4}}$$

is assumed to be a bivariate normal with a covariance matrix defined as:

(5) $$COV\left(\begin{array}{c} Z_{i_{1}j_{1}k_{1}}-Z_{i_{2}j_{2}k_{2}}\\ Z_{i_{3}j_{3}k_{3}}-Z_{i_{4}j_{4}k_{4}} \end{array}\right)=\left(\begin{array}{cc} 1 & \rho {\;}^{*}\\ \rho {\;}^{*} & 1 \end{array}\right).$$

$\rho^{*}$ will vary depending on the correlations between the pairs of transformed prediction scores; for scenarios where all scores originate from different family-level observations, $\rho^{*}$ is defined as 0. The assumption of bivariate normality is useful for calculating the variance of $\hat{\theta_{3}}$, as described in the Appendix.

Assuming that there is a location shift (μ≠0) in the transformed prediction scores between those who did and did not experience the event, the expectation of $\hat{\theta_{3}}$ is a composite of three $\theta^{\prime }s$ as defined below.

(6) E(θ3^)=θs∑i=1I∑j=1Jifijgij+θf∑i=1I∑j1=1Ji∑j2≠j1Jifij1gij2+θ[F×G−(∑i=1I∑j=1Jifijgij+∑i=1I∑j1=1Ji∑j2≠j1Jifij1gij2)]F×G

where

$\theta_{s}=P$ (a subunit that does not have an event has a lower prediction score than a subunit that has an event from the same person) = $\Phi (\mu ∕\sqrt{2(1-\rho_{s})}$) (e.g., comparing risk scores from two eyes of the same person).

$\theta_{f}=P$ (a subunit that does not have an event has a lower prediction score than a subunit that has an event from a different person in the same family) = $\Phi (\mu ∕\sqrt{2(1-\rho_{f})}$).

θ=P (a subunit that does not have an event has a lower prediction score than subunit that has an event from a person in a different family) = $\Phi (\mu ∕\sqrt{2})$.

$\rho_{s}$ = Correlation between transformed prediction scores for subunits of a person.

$\rho_{f}$ = Correlation between transformed prediction scores for subunits of two different persons within the same family.


$$\;f_{ij}=\sum_{k=1}^{K_{ij}}\delta_{ijk}\;\;g_{ij}=\sum_{k=1}^{K_{ij}}(1-\delta_{ijk})\;\;F=\sum_{i=1}^{I}\sum_{j=1}^{J_{i}}\sum_{k=1}^{K_{ij}}\delta_{ijk}\;G=\sum_{i=1}^{I}\sum_{j=1}^{J_{i}}\sum_{k=1}^{K_{ij}}(1-\delta_{ijk})$$

Assuming that there is no location shift in the transformed prediction scores (μ=0), the above expectation is equal to 0.5. To calculate the variance of $\hat{\theta_{3}}$, $\hat{\theta_{3}}$ is first respecified as:

$$\hat{\theta_{3}}=\frac{A+B+C}{F\times G}$$

where $A=\sum_{i=1}^{I}\sum_{j=1}^{J_{i}}\sum_{k_{1}=1}^{K_{ij}}\sum_{k_{2}\neq k_{1}}^{K_{ij}}(1-\delta_{ijk_{1}})\delta_{ijk_{2}}I(S_{ijk_{1}}-S_{ijk_{2}})$; number of subunit comparisons within a person. $B=\sum_{i=1}^{I}\sum_{j_{1}=1}^{J_{i}}\sum_{j_{2}\neq j_{1}}^{J_{i}}\sum_{k_{1}=1}^{K_{ij_{1}}}\sum_{k_{2}=1}^{K_{ij_{2}}}(1-\delta_{ij_{1}k_{1}})\delta_{ij_{2}k_{2}}I(S_{ij_{1}k_{1}}-S_{ij_{2}k_{2}})$; number of subunit comparisons between members of the same family. $C=\sum_{i_{1}=1}^{I}\sum_{i_{2}\neq i_{1}}^{I}\sum_{j_{1}=1}^{J_{i_{1}}}\sum_{j_{2}=1}^{J_{i_{2}}}\sum_{k_{1}=1}^{K_{i_{1}j_{1}}}\sum_{k_{2}=1}^{K_{i_{2}j_{2}}}(1-\delta_{i_{1}j_{1}k_{1}})\delta_{i_{2}j_{2}k_{2}}I(S_{i_{1}j_{1}k_{1}}-S_{i_{2}j_{2}k_{2}})$; number of subunit comparisons between members of different families.

Then,

(7) $$VAR(\hat{\theta_{3}})=\frac{1}{F^{2}\times G^{2}}[VAR(A)+VAR(B)+VAR(C)+2COV(A,B)+2COV(A,C)+2COV(B,C)]$$

The Appendices (Appendix B) provide details of the derivation of the individual terms in Equation (7).

## 3. Estimation of Confidence Limits for $\hat{\theta_{3}}$

Since $\theta_{3}$ is bounded by 0 and 1, the confidence interval surrounding the estimate of this parameter should be bounded similarly. To ensure this, a probit transformation can be applied to $\theta_{3}$ as $\omega_{3}=\Phi^{-1}(\theta_{3})$ where $\Phi^{-1}$ is the inverse of the cumulative standard normal probability distribution. Under the assumption of normality of $\hat{\omega_{3}}$, a 100(1−α)% confidence interval for $\omega_{3}$ is:

(8) $$\left[\hat{\omega_{3}}-z_{\alpha ∕2}SE(\hat{\omega_{3}}),\hat{\omega_{3}}+z_{\alpha ∕2}SE(\hat{\omega_{3}})\right]$$

where $z_{\alpha ∕2}$ is the 100(1−α∕2)th percentile of the standard normal distribution. To find $SE(\hat{\omega_{3}})$, the delta method is applied to $\hat{\theta_{3}}$, which has a known variance (Equation (7)).

$$\;\sqrt{n}(\hat{\theta_{3}}-\theta_{3})\to N[0,VAR({\hat{\theta }}_{3})]\;\sqrt{n}[\Phi^{-1}(\hat{\theta_{3}})-\Phi^{-1}(\theta_{3})]\to N(0,\frac{VAR(\hat{\theta_{3}})\;\;}{{\left\{\phi [\Phi^{-1}(\theta_{3})]\right\}}^{2}})$$

where ϕ is the standard normal probability density. Therefore, $SE(\hat{\omega_{3}})=\frac{SE(\hat{\theta_{3}})}{|\phi [\Phi^{-1}(\theta_{3})]|}$.

A back-transformation of Equation (8) using the cumulative standard normal probability distribution can then be used to obtain the desired confidence interval for $\theta_{3}$.


(9) $$\left\{\Phi [\hat{\omega_{3}}-z_{\alpha ∕2}SE({\hat{\omega }}_{3})],\Phi [\hat{\omega_{3}}+z_{\alpha ∕2}SE(\hat{\omega_{3}})]\right\}$$

## 4. Incorporation of Ties

As stated in Section 2, the inferential procedures for estimating $\hat{\theta_{3}}$ and its confidence limits require that no prediction scores be tied. For prediction scores generated by models having multiple continuous, finely measured predictors, the number of tied subunit prediction scores will likely be negligible, and a single random tie-breaking method may be adopted. For scenarios with a moderate number of ties, this approach will be inefficient and may produce biased results. With the presence of many ties in the data, repeated random tie-breaking followed by a pooling of estimates may be adopted [9] . This procedure is outlined as follows:

1. For each prediction score that is tied with at least one other prediction score, add to it a randomly generated value from a continuous uniform probability distribution defined from −Δ to Δ where Δ is a small number. As an example, Δ may be chosen to be a number more precise than the observed prediction scores (e.g., if prediction scores are measured to the hundredths digit, then 0.001 could be used for Δ). Call this new set of prediction scores (all unique prediction scores and the formerly tied scores) $S_{\;\;ijk}^{\ell }$, where ℓ indexes the set of prediction scores after ties are broken, of which there will be L in total.
2. Using $S_{\;\;ijk}^{\ell }$, calculate $\hat{\theta_{3}}$ and $VAR(\hat{\theta_{3}})$ as usual. Call these estimates ${\hat{\theta_{3}}}^{\ell }$ and ${VAR(\hat{\theta_{3}})}^{\ell }$. As shown in Section 3, apply the probit transformation to ${\hat{\theta_{3}}}^{\ell }$ and call this transformed estimate ${\omega_{3}}^{\ell }$.
3. Repeat steps 1 and 2 L times. Thus, there will be L estimates of $\hat{\theta_{3}}$, $VAR(\hat{\theta_{3}})$ and $\omega_{3}$.
4. Calculate ${\omega_{3}}^{*}$ as $\frac{1}{L}\sum_{\ell =1}^{L}\omega_{3}^{\;\;\ell }$, ${\hat{\theta_{3}}}^{{}^{*}}$ as $\frac{1}{L}\sum_{\ell =1}^{L}{\hat{\theta_{3}}}^{{}^{\ell }}$, and ${VAR(\hat{\theta_{3}})}^{*}$ as $\frac{1}{L}\sum_{\ell =1}^{L}{VAR(\hat{\theta_{3}})}^{{}^{\ell }}+(1+L^{-1})\frac{1}{L-1}\sum_{\ell =1}^{L}{\left({\hat{\theta_{3}}}^{{}^{\ell }}-{\hat{\theta_{3}}}^{{}^{*}}\right)}^{{}^{{}^{2}}}$

Ten iterations of tie breaking (L=10) were found to be reasonable, but if computing power allows, users may run more and assess the stability of their results. ${\hat{\theta_{3}}}^{{}^{*}}$ is interpreted as the AUC after accounting for ties and $\omega_{3}^{\;\;{}^{*}}$, and ${VAR(\hat{\theta_{3}})}^{*}$ are used in place of $\omega_{3}$ and $VAR(\hat{\theta_{3}})$, respectively, for calculating the confidence interval defined in Section 3.

## 5. Simulation Studies

The validity of the point and interval estimates given in Equations (4) and (9) was assessed through simulation. Five scenarios were examined and are described in detail in their respective sections below. To simulate data, prediction scores were derived from the framework of a normal linear mixed model, specified as:

(10) $$Y_{ijk}=B_{1}event_{ijk}+u_{i}+v_{ij}+e_{ijk}$$

with a fixed effect $B_{1}$ for the dichotomous event status ${event}_{ijk}$, normally distributed random intercepts for family ($u_{i}$) and person ($v_{ij}$), and a normally distributed residual for subunit ($e_{ijk}$), all with mean zero. Event status was randomly generated from a Bernoulli distribution with a predefined probability (i.e., prevalence), which was varied throughout the simulation scenarios. The regression coefficient associated with the fixed effect for event status ($B_{1}$) was 1.5, implying that having the event is associated with a higher prediction score. The variance of the family random effect ($u_{i}$) was 5.0, of person ($v_{ij}$) was 3.0, and of subunit ($e_{ijk}$) was 2.0. Given this, the intra-class correlation at the person level was 0.50 and at the subunit level was 0.80. Due to the above simulation parametrizations, all simulation settings had μ=0.474, $\theta_{s}=0.773$, $\theta_{f}=0.682$, and θ=0.631. The five simulation scenarios examined the effects of:

1. Varying the prevalence of event status,
2. accounting for tied prediction scores using the method outlined in Section 4,
3. having a variable number of persons per family, reflecting the 2012 US Census data,
4. creating unbalanced data at the subunit level through the random deletion of subunit measurements, and
5. introducing random variation to the $S_{ijk}$ prediction scores, which are assumed to be measured without error in Equation (2).

For each parametrization of each scenario, 5000 datasets were simulated with a prespecified number of families, persons within families (except in Scenario 3), and subunits. $\hat{\theta_{3}}$ calculated using Equation (4) (averaged over simulations) was compared to the expectation of $\hat{\theta_{3}}$ calculated using Equation (6) to assess bias ($\text{mean}[\hat{\theta_{3}}]$ minus $E[\hat{\theta_{3}}]$). The variance of $\hat{\theta_{3}}$ was examined by forming the ratio of the theoretical variance of $\hat{\theta_{3}}$ calculated using Equation (7), to the empirical variance, which was calculated by taking the variance of the estimated $\hat{\theta_{3}}$ across simulations (theoretical var $\hat{\theta_{3}}$/ empirical var $\hat{\theta_{3}}$). Lastly, the actual confidence interval coverage was measured for the proposed approach, the Obuchowski [6] and Delong [1] confidence interval methods, and the two-level approach of Rosner, Qiu, and Lee [8].

### 5.1. Scenario 1

In Scenario 1, the number of persons per family and the number of subunits per person were constant, and the event status for every subunit was independently assigned using a Bernoulli distribution with a probability of 0.50 or 0.25. Results are listed in Table 1. Across these simulation settings, the mean bias is 0.000, the mean variance ratio is 0.970, and the mean 95% CI coverage is 0.951; for the Obuchowski method, the mean 95% CI coverage is 0.935, for the DeLong method it is 0.938, and for the Rosner method it is 0.941. For the first parameterization in Scenario 1 (20 families, 2 persons, and 50% event prevalence), the actual confidence interval coverage was calculated to assess the viability of using a percentile bootstrap approach with 100 bootstrap samples by applying the same simulation methodology as described above. The percentile bootstrapping approach has a 95% CI coverage of 0.935.

### 5.2. Scenario 2

For Scenario 2, all the above design considerations were maintained, except that prediction scores were binned according to ventiles (20 bins), deciles (10 bins), and quintiles (5 bins) to create tied data. The analysis then proceeded as outlined in Section 4. Results are listed in Table 2. Across these simulation settings, the mean bias is −0.003, the mean-variance ratio is 0.955, and the mean 95% confidence interval coverage is 0.949.

### 5.3. Scenario 3

The design of Scenario 3 was similar to that of Scenario 1, except the number of persons within a family was allowed to vary randomly according to the empirical distribution of the number of children per family (in families with children) from the 2012 United States census data [13]. Families could have up to 10 children with a probability of 0.48 for one child, 0.36 for two children, 0.15 for three children, and 0.03 for four children with progressively smaller probabilities for more. Based on Table 3, the mean bias is 0.001, the mean-variance ratio is 0.965, and the mean 95% confidence interval coverage is 0.949; for the Obuchowski method, the mean 95% CI coverage is 0.939, for the DeLong method it is 0.936, and for the Rosner method it is 0.940.

### 5.4. Scenario 4

Scenario 4 was a modification of Scenario 1, where missing data at the subunit level was introduced. In each simulation, 10% of the persons were randomly selected to have one missing subunit. These missing observations were deleted rather than imputed. Based on Table 4, the mean bias is 0.000, the mean-variance ratio is 0.981, and the mean 95% confidence interval coverage is 0.952; for the Obuchowski method, the mean 95% CI coverage is 0.943, for the DeLong method it is 0.942, and for the Rosner method it is 0.944.

### 5.5. Scenario 5

In Scenario 5, the same setting as used in Scenario 1 was examined, however random normal measurement error (mean set to 0 and standard deviation varied was applied to the $S_{ijk}$ prediction scores (defined as $Y_{ijk}$ in the simulation setting). This was performed to assess the robustness of the proposed method to variability in the $S_{ijk}$ values that are assumed to be measured without error. Based on Table 5, the mean bias is −0.001, the mean-variance ratio is 0.954, and the mean 95% CI coverage is 0.946.

Based on the simulations, the bias in the point estimate of $\hat{\theta_{3}}$ is negligible, and the coverage of the 95% confidence intervals is appropriate.

## 6. Example with Longitudinal Blood Pressure Data

To illustrate the proposed methodology, a sample of longitudinal blood pressure data collected across four timepoints from 1954 to 1968 in South Wales was analyzed [5]. Two regions, the Rhondda Fach and the Vale of Glamorgan, were sampled through the random selection of propositi older than 5 years, whose first-degree relatives were then included in the study along with the propositi. For this illustration, only individuals older than 30 at baseline and only those who were siblings of the propositi or the propositus himself/herself were included. Additionally, only timepoints with blood pressure data available were included. Hypertension was defined as a systolic blood pressure ≥ 140 mmHg, a diastolic blood pressure ≥ 90 mmHg, or a self-reported use of blood pressure medication. A summary of the sample sizes at different levels within the clustered longitudinal structure of the study is presented in Table S1, and descriptive statistics for the baseline visit are displayed in Table S2 of the Appendix.

A generalized linear mixed model with a logit link and Bernoulli response was fit for hypertension (binary, time-varying), with age (continuous, time-varying), BMI (continuous, time-varying), sex (binary, time-invariant), and region (binary, time-invariant) as predictors of interest. Due to a large quantity of missing BMI data, ten rounds of multiple imputations were applied to this variable. Age and BMI were specified using restricted cubic splines with five knots having locations at the 5th, 27.5th, 50th, 72.5th, and 95th percentiles [4]. The inclusion of interactions between age and sex and BMI and sex were assessed using the Akaike Information Criterion (AIC) and found not to improve predictive ability. A family-level random intercept and a sibling random intercept within families, both assumed to be normally distributed, were included. The Kenward–Roger degrees of freedom and fixed effect standard error adjustments were used, and the model was estimated with residual pseudo-likelihood and optimized using Newton–Raphson with ridging in PROC GLIMMIX (SAS 9.4). Within each imputation dataset, the final sample size was 3143 visits (58% hypertensive) with 966 siblings and 319 families, a mean of 3.03 siblings per family (minimum = 1, maximum = 12), and a mean of 3.25 visits per sibling. The final model is given by:

$$\text{ln}\left(\frac{p(hypertension_{ijk})|u_{i},v_{ij}}{1-p(hypertension_{ijk})|u_{i},v_{ij}}\right)=\beta_{0}+\beta_{1-4}{\text{age spline}}_{ijk}+\beta_{5-8}{\text{BMI spline}}_{ijk}+\beta_{9}{\text{sex}}_{ij}+\beta_{10}{\text{region}}_{ij}+u_{i}+v_{ij}$$

where $u_{i}$ is a family-level random intercept and $v_{ij}$ is a sibling-level random intercept.

Due to the use of flexible continuous predictor parameterizations, observed associations from the model fit to the complete dataset have been presented using categorical representations in Table 6—the impact of multiple imputation on inference is accounted for through Rubin’s rules in this table [9]. As seen in Table 6, an increase in age or BMI is strongly associated with higher odds of hypertension. The estimated variance of the random intercept for family was 0.51 (Wald p-value < 0.001) and for sibling was 1.67 (Wald p-value < 0.0001).

Using the estimated parameters of this model, the unconditional (i.e., not conditional on the random intercepts) predicted log odds for each observation in the complete dataset were calculated. By applying the proposed methodology, the AUC was estimated to be 0.76 (μ=0.36, $\theta_{s}=0.70$, $\theta_{f}=0.63$, θ=0.60) with a 95% confidence interval of 0.72 to 0.79. A naïve analysis that does not account for the clustered nature of the data produces the same AUC with a narrower 95% confidence interval of 0.74–0.77 (DeLong method [1]), and an analysis that accounts for clustering at only the participant level produces the same AUC as well, but also results in a narrower 95% confidence interval of 0.73–0.78 (Obuchowski method [6]). Given that multiple imputation was used during model fitting, an approach similar to that outlined in Section 4 was applied to the AUC estimates and confidence intervals to accurately account for the variance introduced by the multiple imputation procedure used for missing BMI data.

## 7. Example with Age-Related Macular Degeneration Data Clustered within Person and Family

The previous example illustrated how confidence intervals for the AUC based on longitudinal data can be produced using the proposed methodology; in this example, data measuring the progression of age-related macular degeneration (AMD) at the individual eye-level but clustered within patients (up to two eyes per person) and families was explored. The data used are from a subset of eyes from patients from the Seddon Longitudinal Cohort Study who did not present with evidence of advanced macular degeneration at a baseline visit and were followed for five years [10]. The sample sizes at each level of clustering are presented in Table S3, and descriptive statistics for the entire sample are presented in Tables S4 and S5 of the Appendix.

A generalized linear mixed model with a logit link and Bernoulli response was fit for eye-level progression (binary) with age (categorical), sex (binary), smoking status (categorical), education (binary), BMI (categorical), and a baseline measure of AMD severity known as the Clinical Age Related Maculopathy Grading System (CARMS) grade (categorical; defined on a scale from 1 to 5, with grades 4 and 5 indicating subtypes of advanced AMD, but only values 1–3 at baseline are present in these example data) as predictors [11]. Race was not included since AMD is much less common in nonwhites, and there were few nonwhites in this cohort. A family-level random intercept and a patient-level random intercept, both assumed to be normally distributed, were included. The Kenward–Roger degrees of freedom and fixed effect standard error adjustments were applied, and the model was estimated by residual pseudo-likelihood and optimized using Newton–Raphson with ridging in PROC GLIMMIX (SAS 9.4). No missing data were present. The final model is:

$$\text{ln}\left(\frac{p(progression_{ijk})|u_{i},v_{ij}}{1-p(progression_{ijk})|u_{i},v_{ij}}\right)=\beta_{0}+\beta_{1}{\text{age}}_{ij}+\beta_{2}{\text{sex}}_{ij}+\beta_{3}{\text{past smoker}}_{ij}+\beta_{4}{\text{current smoker}}_{ij}+\beta_{5}{\text{education}}_{ij}+\;\;\beta_{6}\text{BMI}\;2_{ij}+\beta_{7}\text{BMI}\;3_{ij}+\beta_{8}\text{CARMS}\;2_{ijk}+\beta_{9}\text{CARMS}\;3_{ijk}+u_{i}+v_{ij}$$

where $u_{i}$ is a family-level random intercept and $v_{ij}$ is a person-level random intercept.

The sample size was 1396 eyes (8% with progression), 741 patients, and 325 families. The results from the model fit using the complete dataset are presented in Table 7 where it can be seen that age and CARMS grade were the strongest predictors of progression. The estimated variance of the random intercept for family was 1.04 (Wald p-value = 0.027) and for person was 0.71 (Wald p-value = 0.09).

Using the estimated parameters of this model, the unconditional (i.e., not conditional on the random intercepts) predicted log odds for each observation in the complete dataset were calculated. There were many ties in the resulting predictions, so the previously outlined tie-breaking approach was used. By applying the proposed methodology, the AUC was estimated to be 0.88 (μ=1.21, $\theta_{s}=0.99$, $\theta_{f}=0.85$, θ=0.80) with a 95% confidence interval of 0.82–0.92. A correlation-naïve analysis that does not account for the hierarchical nature of the data produces the same AUC with a narrower 95% confidence interval of 0.85–0.90 (DeLong method [1]), and an analysis that accounts for clustering at only the participant level produces the same AUC as well, but also results in a narrower 95% confidence interval of 0.85–0.91 (Obuchowski method [6]).

## 8. Discussion

This investigation described how the extended Mann–Whitney U statistic, when paired with normally transformed prediction scores, can be applied to three-level hierarchical data to calculate an AUC statistic with a corresponding confidence interval. The AUC is calculated based on prediction scores from a three-level generalized linear mixed model with a logit link and Bernoulli distribution. Data may originate from a clustered setting or a longitudinal setting and cluster sizes are allowed to vary, but the correlation structure must be exchangeable. Simulation studies showed that the coverage of the 95% confidence interval around the AUC is appropriate, even in unbalanced settings with small cluster sizes and a small number of independent units (i.e., families).

In the two examples provided, the data were not divided into a training and testing set as this was not relevant to the illustration of the methodology. In practice, this technique or cross-validation could be used to obtain a validated measure of AUC. Cross-validation can be used by calculating the variance and AUC of the holdout sets of data and then combining them through the use of Rubin’s rules [9].

In the presentation of the two-level approach, prediction scores were assumed to be generated through a generalized estimating equation (GEE) model [8]. A three-level hierarchical data is traditionally modeled using generalized linear mixed models, and so this technique is used here in lieu of GEE. If a marginal model rather than a subject-specific model is desired, GEE may still be used, assuming that a method supporting three-levels of data dependency is applied.

Two important assumptions deserve further attention. First, Equation (5) assumes bivariate normality of normal random variables. The authors believe that this assumption is appropriate given that an examination of 3D histograms of the presented example data and data simulation scenarios did not violate this assumption. Second, this methodology assumes that the correlation structure of the response data is exchangeable. This is typically valid in data settings where correlation is induced by observations being nested within groups but is often inappropriate with longitudinal data. The blood pressure example (Section 6) includes longitudinal data, but due to model nonconvergence, it was not possible to assess the utility of complex correlation structures. As noted in Fitzmaurice, Laird, and Ware [2], it is often difficult to specify correlation structures that are more flexible than exchangeable ones in generalized linear mixed models with binary responses. Regardless, users of the proposed methodology should attempt to determine if exchangeability is a fair assumption. In addition to assessing the correlation structure, users should also be cognizant of the presence of informative cluster sizes, which may lead to bias in the results from the proposed methodology. The authors report that there are no competing interests to declare. For users interested in applying the proposed method to construct valid confidence intervals for the AUC in three-level settings, R code is available in the Supplementary Materials.

## Supplementary Material

Appendix

## Figures and Tables

**Table 1.** Simulation study with event prevalence of 0.50 or 0.25 (n=5000 simulations).

								Actual 95% CI Coverage			
Families	Persons	Subunits	Event Prevalence	$\text{Mean}(\hat{\theta_{3}})$	$E[\hat{\theta_{3}}]$	Bias	Variance Ratio	Proposed	Obuchowski	Delong	Rosner
20	2	2	0.50	0.635	0.634	0.001	0.989	0.958	0.935	0.926	0.943
50	2	2	0.50	0.633	0.633	0.000	0.920	0.947	0.943	0.943	0.944
100	2	2	0.50	0.632	0.632	0.000	0.951	0.947	0.945	0.944	0.944
20	3	2	0.50	0.634	0.634	0.000	0.996	0.955	0.934	0.924	0.940
50	3	2	0.50	0.632	0.633	−0.001	0.985	0.951	0.939	0.945	0.942
100	3	2	0.50	0.632	0.632	0.001	1.007	0.957	0.949	0.946	0.938
20	2	2	0.25	0.633	0.634	−0.001	0.964	0.956	0.934	0.924	0.945
50	2	2	0.25	0.633	0.633	0.000	0.965	0.948	0.943	0.942	0.949
100	2	2	0.25	0.632	0.632	0.000	0.979	0.951	0.944	0.947	0.939
20	3	2	0.25	0.635	0.634	0.001	0.931	0.947	0.933	0.924	0.939
50	3	2	0.25	0.631	0.633	−0.002	0.977	0.950	0.944	0.948	0.937
100	3	2	0.25	0.632	0.632	0.000	0.978	0.950	0.939	0.948	0.943
Mean				0.633	0.633	0.000	0.970	0.951	0.935	0.938	0.941

Variance ratio is theoretical/empirical variance; actual 95% CI coverage is presented for the proposed method, Obuchowski’s method [6], DeLong’s method [1], and Rosner’s method [8].

**Table 2.** Simulation study with tied prediction scores (n=5000 simulations).

Families	Persons	Subunits	Bins	$\mathrm{Mean}(\hat{\theta_{3}})$	$E[\hat{\theta_{3}}]$	Bias	Variance Ratio	Actual 95% CI Coverage
20	2	2	5	0.627	0.634	−0.007	0.968	0.953
50	2	2	5	0.626	0.633	−0.007	0.955	0.943
20	2	2	10	0.633	0.634	−0.001	0.955	0.952
50	2	2	10	0.631	0.633	−0.002	0.944	0.945
20	2	2	20	0.635	0.634	0.001	0.963	0.954
50	2	2	20	0.632	0.633	−0.001	0.946	0.944
Mean				0.631	0.634	−0.003	0.955	0.949

Variance ratio is theoretical/empirical variance; bins is percentile bins; event prevalence is 0.50 for all scenarios; actual 95% CI coverage is presented for the proposed method only, as the tie-breaking approach discussed in Section 4 is required.

**Table 3.** Simulation study with variable family size following the 2012 United States census (n=5000 simulations).

							Actual 95% CI Coverage			
Families	Subunits	Event Prevalence	$\text{Mean}(\hat{\theta_{3}})$	$E[\hat{\theta_{3}}]$	Bias	Variance Ratio	Proposed	Obuchowski	DeLong	Rosner
50	2	0.25	0.633	0.633	0.000	0.988	0.952	0.938	0.932	0.939
50	2	0.50	0.634	0.633	0.001	0.941	0.946	0.940	0.940	0.940
Mean			0.634	0.633	0.001	0.965	0.949	0.939	0.936	0.940

Variance ratio is theoretical/empirical variance; actual 95% CI coverage is presented for the proposed method, Obuchowski’s method [6], DeLong’s method [1], and Rosner’s method [8].

**Table 4.** Simulation study with 10% of persons having a single ineligible subunit (n=5000 simulations).

							Actual 95% CI Coverage			
Families	Persons	Event Prevalence	$\text{Mean}(\hat{\theta_{3}})$	$E[\hat{\theta_{3}}]$	Bias	Variance Ratio	Proposed	Obuchowski	DeLong	Rosner
50	2	0.50	0.632	0.633	−0.001	0.944	0.948	0.947	0.948	0.949
50	3	0.50	0.633	0.633	0.000	1.005	0.954	0.942	0.940	0.940
50	2	0.25	0.632	0.633	−0.001	0.961	0.950	0.941	0.940	0.945
50	3	0.25	0.633	0.633	0.000	1.013	0.955	0.942	0.941	0.940
Mean			0.633	0.633	0.000	0.981	0.952	0.943	0.942	0.944

Variance ratio is theoretical/empirical variance; in each scenario, 90% of persons had two subunits and 10% had one subunit; actual 95% CI coverage is presented for the proposed method, Obuchowski’s method [6], DeLong’s method [1], and Rosner’s method [8].

**Table 5.** Simulation study with random normal error (mean = 0) introduced to the $S_{ijk}$ prediction scores.

Families	Persons	Subunit	Event Prevalence	Normal Random Error SD	$\text{Mean}(\hat{\theta_{3}})$	$E[\hat{\theta_{3}}]$	Bias	Variance Ratio	95% CI Coverage
50	2	2	0.50	0.1	0.632	0.633	−0.001	0.918	0.946
50	3	2	0.50	0.1	0.633	0.633	0.000	0.982	0.948
50	2	2	0.50	0.25	0.631	0.633	−0.002	0.939	0.945
50	3	2	0.50	0.25	0.633	0.633	0.000	0.993	0.952
50	2	2	0.50	0.50	0.631	0.633	−0.002	0.959	0.947
50	3	2	0.50	0.50	0.630	0.633	−0.003	0.935	0.937
Mean					0.632	0.633	−0.001	0.954	0.946

Variance ratio is theoretical/empirical variance; SD = standard deviation.

**Table 6.** The association between variables of interest and hypertension using categorized specifications of continuous predictors; random intercept variances; and AUC in the complete South Wales dataset (n visits = 3143, n siblings = 966, and n families = 319).

Variable	Odds Ratio	95% Confidence Interval	p-Value
Sex, reference = female	0.75	(0.58, 0.98)	0.037
Age, reference = (30–39.9)			<0.0001
(40–49.9)	1.43	(1.07, 1.92)	
(50–59.9)	3.01	(2.19, 4.14)	
(60–69.9)	8.60	(5.88, 12.57)	
≥70	17.10	(10.49, 27.89)	
BMI, reference < 25			<0.0001
(25–29.9)	1.74	(1.36, 2.21)	
≥30	3.88	(2.63, 5.73)	
Region, reference = Vale of Glamorgan	0.76	(0.57, 1.01)	0.06
Family random intercept variance	0.51 (Wald p-value < 0.001) *		
Person random intercept variance	1.67 (Wald p-value < 0.0001) *		
AUC (proposed methodology)	0.76 (95% CI: 0.72 to 0.79)		
DeLong AUC [1]	0.76 (95% CI: 0.74 to 0.77)		
Obuchowski AUC [6]	0.76 (95% CI: 0.73 to 0.78)		

* Model-based Wald test of nonzero variance.

**Table 7.** The association between predictors of interest and eye-level progression to advanced age-related macular degeneration (AMD); random intercept variances; and AUC in the complete AMD dataset (1396 eyes [107 eyes progressing within five years], 741 patients, and 325 families).

Variable	Odds Ratio	95% Confidence Interval	p-Value
Age (years), reference = (55–64.9)			0.010
(65–74.9)	0.94	(0.41, 2.15)	
(75–80)	2.43	(1.00, 5.87)	
Sex, reference = female	0.58	(0.31, 1.08)	0.08
Smoking status, reference = never			0.60
Past	1.26	(0.68, 2.35)	
Current	1.64	(0.54, 5.00)	
Education, reference = more than high school	1.28	(0.73, 2.26)	0.39
BMI, reference <25			0.50
(25–29.9)	0.68	(0.36, 1.30)	
≥30	0.85	(0.40, 1.83)	
CARMS grade, reference = 1			<0.0001
2	25.95	(5.76, 116.86)	
3	89.18	(20.91, 380.30)	
Family random intercept variance	1.04 (Wald p-value = 0.027) *		
Person random intercept variance	0.71 (Wald p-value = 0.09) *		
AUC (proposed methodology)	0.88 (95% CI: 0.82 to 0.92)		
DeLong AUC [1]	0.88 (95% CI: 0.85 to 0.90)		
Obuchowski AUC [6]	0.88 (95% CI: 0.85 to 0.91)		

* Model-based Wald test of nonzero variance.

## Data Availability

No new data were created for this study. Data from the relevant original studies are available from the authors upon reasonable request.
