# Supplementary material for: Evaluation of Risk Prediction with Hierarchical Data: Dependency Adjusted Confidence Intervals for the AUC
Source: Stats (Basel). Author manuscript; Available in PMC 2023 Nov 2. (PMC10621602; doi:10.3390/stats6020034)
Supplement: Appendix [file NIHMS1935806-supplement-Appendix.docx]

**Appendix A: Supplementary Tables**

Table S1. Sample sizes for family, sibling, and visit in complete South Wales dataset

| Number of families | 319 |
| --- | --- |
| Number of siblings | 966 |
| Number of siblings per family |  |
| 1 (propositi only) | 100 (31%) |
| 2 | 68 (21%) |
| 3+ | 151 (47%) |
| Number of visits per sibling |  |
| 1 | 99 (10%) |
| 2 | 119 (12%) |
| 3 | 186 (19%) |
| 4 | 562 (58%) |

Table S2. Baseline visit demographic information for the complete South Wales dataset (n = 966)

|  | No Hypertension (n = 437) | Hypertension (n = 529) |
| --- | --- | --- |
| Age (years) |  |  |
| (30 – 39.9) | 208 (47.6%) | 97 (18.3%) |
| (40 – 49.9) | 132 (30.2%) | 113 (21.4%) |
| (50 – 59.9) | 67 (15.3%) | 134 (25.3%) |
| (60 – 69.9) | 22 (5.0%) | 115 (21.7%) |
| ≥ 70 | 8 (1.9%) | 70 (13.2%) |
| Mean (sd) | 42.4 (10.1) | 53.8 (13.6) |
| Sex |  |  |
| Male | 232 (53.1%) | 275 (52.0%) |
| Female | 205 (46.9%) | 254 (48.0%) |
| BMI |  |  |
| < 25 | 192 (63.4%) | 181 (44.8%) |
| [25 – 29.9) | 94 (31.0%) | 147 (36.4%) |
| ≥ 30 | 17 (5.6%) | 76 (18.8%) |
| Not available | 134 | 125 |
| Mean (sd) | 24.0 (3.3) | 26.0 (4.6) |
| Hypertension |  |  |
| Systolic blood pressure |  |  |
| Mean (sd) | 120.7 (9.8) | 159.3 (26.3) |
| Diastolic blood pressure |  |  |
| Mean (sd) | 75.8 (7.7) | 91.2 (11.4 |
| Region |  |  |
| Vale of Glamorgan | 242 (55.4%) | 319 (60.3%) |
| Rhondda Fach | 195 (44.6%) | 210 (39.7%) |

sd: standard deviation; values are frequency (percent) unless otherwise noted. 66 individuals self-reported using hypertension medication and were classified as having hypertension based on this information

Table S3. Sample sizes for eye, patient, and family for the complete age-related macular degeneration dataset

| Number of eyes | 1,396 |
| --- | --- |
| Number of patients | 741 |
| Number of families | 325 |
| Number of eligible patients per family |  |
| 1 | 5 (2%) |
| 2 | 54 (17%) |
| 3+ | 266 (82%) |
| Number of eligible eyes per patient (non-advanced at baseline) |  |
| 1 | 86 (12%) |
| 2 | 655 (88%) |

Table S4. Baseline patient-level demographic information for the complete age-related macular degeneration dataset stratified by progression status over five years of follow-up (n = 741 patients)

|  | No Progression (n = 660) | Any progression (n = 81)^a^ |
| --- | --- | --- |
| Age (years) |  |  |
| (55 – 64.9) | 177 (26.8%) | 11 (13.6%) |
| (65 – 74.9) | 368 (55.8%) | 38 (46.9%) |
| (75 – 80) | 115 (17.4%) | 32 (39.5%) |
| Mean (sd) | 69.04 (6.10) | 72.12 (5.24) |
| Sex |  |  |
| Male | 413 (62.6%) | 42 (51.9%) |
| Female | 247 (37.4%) | 39 (48.2%) |
| Race |  |  |
| White | 655 (99.2%) | 81 (100.0%) |
| Non-white | 5 (0.8%) | 0 (0.0%) |
| Smoking status |  |  |
| Current | 30 (4.6%) | 6 (7.4%) |
| Past | 361 (54.7%) | 43 (53.1%) |
| Never | 269 (40.8%) | 32 (39.5%) |
| Education |  |  |
| High school or less | 270 (40.9%) | 41 (50.6%) |
| More than high school | 390 (59.1%) | 40 (49.4%) |
| BMI |  |  |
| < 25 | 209 (31.7%) | 32 (39.5%) |
| [25 – 29.9) | 299 (45.3%) | 28 (34.6%) |
| ≥ 30 | 152 (23.0%) | 21 (25.9%) |
| Number of eligible eyes |  |  |
| 1 (other eye advanced  at baseline) | 58 (8.8%) | 28 (34.6%) |
| 2 | 602 (91.2%) | 53 (65.4%) |

sd: standard deviation; values are frequency (percent) unless otherwise noted

a. progression in either eye

Table S5. Baseline eye-level information for the complete age-related macular degeneration dataset (n = 1,396 eyes)

|  | No progression (n = 1,289) | Progression (n = 107) |
| --- | --- | --- |
| Baseline CARMS grade |  |  |
| 1 | 756 (58.7%) | 2 (1.9%) |
| 2 | 262 (20.3%) | 21 (19.6%) |
| 3 | 271 (21.0%) | 84 (78.5%) |

**Appendix B: Derivation of AUC variance**

1. Notation and Important Equations

For clarity, index $i$will be referred to as families, index$j$will be referred to as persons, and index$k$will be referred to as subunits.

$$\hat{\theta_{3}}=\frac{\sum_{i_{1}=1}^{I} \sum_{j_{1}=1}^{J_{i_{1}}} \sum_{k_{1}=1}^{K_{i_{1}j_{1}}} \sum_{i_{2}=1}^{I} \sum_{j_{2}=1}^{J_{i_{2}}} \sum_{k_{2}=1}^{K_{i_{2}j_{2}}} (1-\delta_{i_{1}j_{1}k_{1}})\delta_{i_{2}j_{2}k_{2}}I(S_{i_{1}j_{1}k_{1}}-S_{i_{2}j_{2}k_{2}})}{\sum_{i_{1}=1}^{I} \sum_{j_{1}=1}^{J_{i_{1}}} \sum_{k_{1}=1}^{K_{i_{1}j_{1}}} \sum_{i_{2}=1}^{I} \sum_{j_{2}=1}^{J_{i_{2}}} \sum_{k_{2}=1}^{K_{i_{2}j_{2}}} (1-\delta_{i_{1}j_{1}k_{1}})\delta_{i_{2}j_{2}k_{2}}}=\frac{A+B+C}{F\times G}$$

where$i_{1}j_{1}k_{1}\neq i_{2}j_{2}k_{2}$

$$\begin{matrix} A:\text{ Comparison of subunits within persons} \\ B:\text{ Comparison of subunits between persons of the same family} \\ C:\text{ Comparison of subunits between persons from different families} \\ F:\text{ Number of subunits that are events} \\ G:\text{ Number of subunits that are non-events} \end{matrix}$$

$$\begin{matrix} \delta_{ijk}=\left\{\begin{matrix} 1\text{ if event} \\ 0\text{ if non-event} \end{matrix}\right. \\ I(S_{1}-S_{2})=\left\{\begin{matrix} 1\text{ if S}_{1}-S_{2}<0 \\ 0\text{ otherwise} \end{matrix}\right. \end{matrix}$$

$$E(\hat{\theta_{3}})=\frac{\theta_{s}\sum_{i=1}^{I} \sum_{j=1}^{J_{i}} f_{ij}g_{ij}+\theta_{f}\sum_{i=1}^{I} \sum_{j_{1}=1}^{J_{i}} \sum_{j_{2}\neq j_{1}}^{J_{i}} f_{ij_{1}}g_{ij_{2}}+\theta\left[ F\times G-\left( \sum_{i=1}^{I} \sum_{j=1}^{J_{i}} f_{ij}g_{ij}+\sum_{i=1}^{I} \sum_{j_{1}=1}^{J_{i}} \sum_{j_{2}\neq j_{1}}^{J_{i}} f_{ij_{1}}g_{ij_{2}} \right) \right]}{F\times G}$$

$$\begin{matrix} f_{ij}=\sum_{k=1}^{K_{ij}} \delta_{ijk} \\ g_{ij}=\sum_{k=1}^{K_{ij}} (1-\delta_{ijk}) \\ f_{i}=\sum_{j=1}^{J_{i}} \sum_{k=1}^{K_{ij}} \delta_{ijk} \\ g_{i}=\sum_{j=1}^{J_{i}} \sum_{k=1}^{K_{ij}} \left(1-\delta_{ijk}\right) \\ \underline{fg}_{i}=\sum_{j=1}^{J_{i}} f_{ij}g_{ij} \\ N=f+g \\ n_{i}=f_{i}+g_{i} \\ n_{ij}=f_{ij}+g_{ij} \\ \theta_{s}=P(S_{ijk_{1}}<S_{ijk_{2}}|\delta_{ijk_{1}}=0,\delta_{ijk_{2}}=1) \\ \theta_{f}=P(S_{ij_{1}k_{1}}<S_{ij_{2}k_{2}}|\delta_{ij_{1}k_{1}}=0,\delta_{ij_{2}k_{2}}=1,j_{1}\neq j_{2}) \\ \theta=P(S_{i_{1}j_{1}k_{1}}<S_{i_{2}j_{2}k_{2}}|\delta_{i_{1}j_{1}k_{1}}=0,\delta_{i_{2}j_{2}k_{2}}=1,i_{1}\neq i_{2}) \end{matrix}$$

$$\begin{matrix} \rho_{s}=\text{Correlation between prediction score subunits within a person} \\ \rho_{f}=\text{Correlation between prediction score subunits from two different persons } \\ \text{ within the same family} \end{matrix}$$

$$VAR(\hat{\theta_{3}})=\frac{1}{F^{2}\times G^{2}}\left[ VAR(A)+VAR(B)+VAR(C)+2COV(A,B)+2COV(A,C)+2COV(B,C) \right]$$

2. *VAR(A)*

$$A=\sum_{i=1}^{I} A_{i}$$

$$A_{i}=\sum_{j=1}^{J_{i}} \sum_{k_{1}=1}^{K_{ij}} \sum_{k_{2}=1}^{K_{ij}} (1-\delta_{ijk_{1}})\delta_{ijk_{2}}I(S_{ijk_{1}}-S_{ijk_{2}})$$

$$A_{i}=\sum_{j=1}^{J_{i}} A_{ij}$$

$$A_{ij}=\sum_{k_{1}=1}^{K_{ij}} \sum_{k_{2}=1}^{K_{ij}} (1-\delta_{ijk_{1}})\delta_{ijk_{2}}I(S_{ijk_{1}}-S_{ijk_{2}})$$

$$VAR(A)=\sum_{i=1}^{I} VAR(A_{i})$$

$VAR(A_{i})=\sum_{j=1}^{J_{i}} VAR(A_{ij})$ (1)

Note that $\sum_{j_{1}=1}^{J_{i}} \sum_{j_{2}\neq j_{1}}^{J_{i}} COV(A_{ij_{1}},A_{ij_{2}})=0$

$$\sum_{j=1}^{J_{i}} VAR(A_{ij})=\sum_{j=1}^{J_{i}} VAR\left[\sum_{k_{1}=1}^{K_{ij}} \sum_{k_{2}=1}^{K_{ij}} (1-\delta_{ijk_{1}})\delta_{ijk_{2}}I(S_{ijk_{1}}-S_{ijk_{2}})\right]=$$

$\sum_{j=1}^{J_{i}} \sum_{k_{A1}=1}^{K_{ij}} \sum_{k_{B1}=1}^{K_{ij}} \sum_{k_{A2}=1}^{K_{ij}} \sum_{k_{B2}=1}^{K_{ij}} (1-\delta_{ijk_{A1}})\delta_{ijk_{B1}}(1-\delta_{ijk_{A2}})\delta_{ijk_{B2}}COV\left[I(S_{ijk_{A1}}-S_{ijk_{B1}}),I(S_{ijk_{A2}}-S_{ijk_{B2}})\right]$ (2)

$$\begin{matrix} VAR(A_{ij})=\theta_{s}(1-\theta_{s})\sum_{j=1}^{J_{i}} \sum_{k_{A1}=k_{A2}}^{K_{ij}} \sum_{k_{B1}=k_{B2}}^{K_{ij}} \left[(1-\delta_{ijk_{A1}})\delta_{ijk_{B1}}\right]^{2}+ \\ \left[\Phi_{2}(\Phi^{-1}(\theta_{s}),\Phi^{-1}(\theta_{s}),0.5)-\theta_{s}^{2}\right]\sum_{j=1}^{J_{i}} \sum_{k_{A1}\neq k_{A2}}^{K_{ij}} \sum_{k_{B1}=k_{B2}}^{K_{ij}} (1-\delta_{ijk_{A1}})\delta_{ijk_{B1}}(1-\delta_{ijk_{A2}})\delta_{ijk_{B1}}+ \\ \left[\Phi_{2}(\Phi^{-1}(\theta_{s}),\Phi^{-1}(\theta_{s}),0.5)-\theta_{s}^{2}\right]\sum_{j=1}^{J_{i}} \sum_{k_{A1}=k_{A2}}^{K_{ij}} \sum_{k_{B1}\neq k_{B2}}^{K_{ij}} (1-\delta_{ijk_{A1}})\delta_{ijk_{B1}}(1-\delta_{ijk_{A1}})\delta_{ijk_{B2}}+ \\ 0 \end{matrix}$$

The following illustrates how $\Phi_{2}\left[\Phi^{-1}(\theta_{s}),\Phi^{-1}(\theta_{s}),0.5\right]$ was derived, where $\Phi_{2}$ denotes a bivariate normal CDF

$$\begin{matrix} COV\left[I(S_{ijk_{A1}}-S_{ijk_{B1}}),I(S_{ijk_{A2}}-S_{ijk_{B1}})\right]= \\ E\left[I(S_{ijk_{A1}}-S_{ijk_{B1}})\times I(S_{ijk_{A2}}-S_{ijk_{B1}})\right]-E\left[I(S_{ijk_{A1}}-S_{ijk_{B1}})\right]\times E\left[I(S_{ijk_{A2}}-S_{ijk_{B1}})\right]= \\ P(S_{ijk_{A1}}-S_{ijk_{B1}}<0,S_{ijk_{A2}}-S_{ijk_{B1}}<0)-\theta_{s}^{2} \end{matrix}$$

The probit transformation (called $H()$ here) is then applied to the prediction scores

$$H(S_{ijk_{A1}})-H(S_{ijk_{B1}})\sim N\left(-\mu,2(1-\rho_{s})\right)$$

$$H(S_{ijk_{A2}})-H(S_{ijk_{B1}})\sim N\left(-\mu,2(1-\rho_{s})\right)$$

$$V_{1}=\frac{H(S_{ijk_{A1}})-H(S_{ijk_{B1}})+\mu}{\sqrt{2(1-\rho_{s})}}\sim N(0,1)$$

$$V_{2}=\frac{H(S_{ijk_{A2}})-H(Z_{ijk_{B1}})+\mu}{\sqrt{2(1-\rho_{s})}}\sim N(0,1)$$

Since $V_{1}$and $V_{2}$ are assumed to be bivariate normal, their CDF is specified as

$$\Phi_{2}(c_{1},c_{2},\rho*)=P\left\{V_{1}\leq c_{1},V_{2}\leq c_{2}|V_{1},V_{2}\sim N\left[\left(\begin{matrix} 0 \\ 0 \end{matrix}\right),\left(\begin{matrix} 1 & \rho* \\ \rho* & 1 \end{matrix}\right)\right]\right\}$$

The parameters of $\Phi_{2}(c_{1},c_{2},\rho*)$can then be re-specified as

$$\Phi_{2}(\Phi^{-1}(\theta_{s}),\Phi^{-1}(\theta_{s}),\rho*)$$

Where $\rho*=COV(V_{1},V_{2})=\frac{\rho_{s}-\rho_{s}-\rho_{s}+1}{2(1-\rho_{s})}=\frac{1}{2}$

The following shows how to simplify the summations for $VAR(A_{ij})$

$$\begin{matrix} \sum_{k_{A1}=k_{A2}}^{K_{ij}} \sum_{k_{B1}=k_{B2}}^{K_{ij}} \left[(1-\delta_{ijk_{A1}})\delta_{ijk_{B1}}\right]^{2}=\sum_{k_{A1}=k_{A2}}^{K_{ij}} (1-\delta_{ijk_{A1}})\sum_{k_{B1}=k_{B2}}^{K_{ij}} \delta_{ijk_{B1}}=\sum_{j=1}^{J_{i}} f_{ij}g_{ij} \\ \sum_{k_{A1}\neq k_{A2}}^{K_{ij}} \sum_{k_{B1}=k_{B2}}^{K_{ij}} (1-\delta_{ijk_{A1}})\delta_{ijk_{B1}}(1-\delta_{ijk_{A2}})\delta_{ijk_{B1}}=\sum_{k_{A1}}^{K_{ij}} \sum_{k_{A2}\neq k_{A1}}^{K_{ij}} (1-\delta_{ijk_{A1}})(1-\delta_{ijk_{A2}})\sum_{k_{B1}}^{K_{ij}} \delta_{ijk_{B1}}=f_{ij}g_{ij}(g_{ij}-1) \\ \sum_{k_{A1}=k_{A2}}^{K_{ij}} \sum_{k_{B1}\neq k_{B2}}^{K_{ij}} (1-\delta_{ijk_{A1}})\delta_{ijk_{B1}}(1-\delta_{ijk_{A1}})\delta_{ijk_{B2}}=\sum_{k_{A1}}^{K_{ij}} (1-\delta_{ijk_{A1}})\sum_{k_{B1}}^{K_{ij}} \sum_{k_{B2}\neq k_{B1}}^{K_{ij}} \delta_{ijk_{B1}}\delta_{ijk_{B2}}=g_{ij}f_{ij}(f_{ij}-1) \end{matrix}$$

$VAR(A_{ij})=\theta_{s}(1-\theta_{s})\sum_{j=1}^{J_{i}} f_{ij}g_{ij}+\left[\Phi_{2}(\Phi^{-1}(\theta_{s}),\Phi^{-1}(\theta_{s}),0.5)-\theta_{s}^{2}\right]\sum_{j=1}^{J_{i}} f_{ij}g_{ij}(f_{ij}+g_{ij}-2)$ (3)

Therefore, $VAR(A)=\theta_{s}(1-\theta_{s})\sum_{i=1}^{I} \sum_{j=1}^{J_{i}} f_{ij}g_{ij}+\left[\Phi_{2}(\Phi^{-1}(\theta_{s}),\Phi^{-1}(\theta_{s}),0.5)-\theta_{s}^{2}\right]\sum_{i=1}^{I} \sum_{j=1}^{J_{i}} f_{ij}g_{ij}(f_{ij}+g_{ij}-2)$ (4)

(4) agrees with $VAR(A)$in the Appendix of Rosner, Qiu, and Lee (2013)

3. *VAR(B)*

$$B=\sum_{i=1}^{I} B_{i}$$

$$VAR(B)=\sum_{i=1}^{I} VAR(B_{i})$$

$$B_{i}=\sum_{j_{1}=1}^{J_{i}} \sum_{j_{2}\neq j_{1}}^{J_{i}} \sum_{k_{1}=1}^{K_{ij_{1}}} \sum_{k_{2}=1}^{K_{ij_{2}}} (1-\delta_{ij_{1}k_{1}})\delta_{ij_{2}k_{2}}I(S_{ij_{1}k_{1}}-S_{ij_{2}k_{2}})$$

$$VAR(B_{i})=VAR\left[\sum_{j_{1}=1}^{J_{i}} \sum_{j_{2}\neq j_{1}}^{J_{i}} \sum_{k_{1}=1}^{K_{ij_{1}}} \sum_{k_{2}=1}^{K_{ij_{2}}} (1-\delta_{ij_{1}k_{1}})\delta_{ij_{2}k_{2}}I(S_{ij_{1}k_{1}}-S_{ij_{2}k_{2}})\right]$$

$VAR(B_{i})=\sum_{y=1}^{5} B_{iy}^{*}$ (5)

$\sum_{y=1}^{5} B_{iy}^{*}$represents the five unique patterns of similarity that can occur when taking the covariance between two score differences, where each score difference is composed of two different subjects (*W≠X≠Y≠Z*).

| *WX* | *YZ* | 0 |
| --- | --- | --- |
| *WX* | *WX* | $B_{i1}^{*}$ |
| *WX* | *WY* | $B_{i2}^{*}$ |
| *WX* | *YX* | $B_{i3}^{*}$ |
| *WX* | *XW* | $B_{i4}^{*}$ |
| *WX* | *YW* | $B_{i5}^{*}$ |
| *WX* | *XY* | $B_{i5}^{*}$ |

$$\begin{matrix} B_{i1}^{*}=\sum_{j_{A1}=j_{A2},j_{A1}\neq j_{B1}}^{J_{i}} \sum_{j_{B1}=j_{B2},j_{A2}\neq j_{B2}}^{J_{i}} \sum_{k_{A1}=1}^{K_{ij}} \sum_{k_{A2}=1}^{K_{ij}} \sum_{k_{B1}=1}^{K_{ij}} \sum_{k_{B2}=1}^{K_{ij}} (1-\delta_{ij_{A1}k_{A1}})\delta_{ij_{B1}k_{B1}}(1-\delta_{ij_{A1}k_{A2}})\delta_{ij_{B1}k_{B2}} \\ COV\left[I(S_{ij_{A1}k_{A1}}-S_{ij_{B1}k_{B1}}),I(S_{ij_{A1}k_{A2}}-S_{ij_{B1}k_{B2}})\right]= \end{matrix}$$

$$\begin{matrix} \sum_{j_{A1}\neq j_{B1}}^{J_{i}} \sum_{k_{A1}=k_{A2}}^{K_{ij}} \sum_{k_{B1}=k_{B2}}^{K_{ij}} (1-\delta_{ij_{A1}k_{A1}})\delta_{ij_{B1}k_{B1}}(1-\delta_{ij_{A1}k_{A1}})\delta_{ij_{B1}k_{B1}}\times\\ COV\left[I(S_{ij_{A1}k_{A1}}-S_{ij_{B1}k_{B1}}),I(S_{ij_{A1}k_{A1}}-S_{ij_{B1}k_{B1}})\right]+ \end{matrix}$$

$$\left(B_{i1,1}^{*}\right)$$

$$\begin{matrix} \sum_{j_{A1}\neq j_{B1}}^{J_{i}} \sum_{k_{A1}\neq k_{A2}}^{K_{ij}} \sum_{k_{B1}=k_{B2}}^{K_{ij}} (1-\delta_{ij_{A1}k_{A1}})\delta_{ij_{B1}k_{B1}}(1-\delta_{ij_{A1}k_{A2}})\delta_{ij_{B1}k_{B1}}\times\\ COV\left[I(S_{ij_{A1}k_{A1}}-S_{ij_{B1}k_{B1}}),I(S_{ij_{A1}k_{A2}}-S_{ij_{B1}k_{B1}})\right]+ \end{matrix}$$

$$\left(B_{i1,2}^{*}\right)$$

$$\begin{matrix} \sum_{j_{A1}\neq j_{B1}}^{J_{i}} \sum_{k_{A1}=k_{A2}}^{K_{ij}} \sum_{k_{B1}\neq k_{B2}}^{K_{ij}} (1-\delta_{ij_{A1}k_{A1}})\delta_{ij_{B1}k_{B1}}(1-\delta_{ij_{A1}k_{A1}})\delta_{ij_{B1}k_{B2}}\times\\ COV\left[I(S_{ij_{A1}k_{A1}}-S_{ij_{B1}k_{B1}}),I(S_{ij_{A1}k_{A1}}-S_{ij_{B1}k_{B2}})\right]+ \end{matrix}$$

$$\left(B_{i1,3}^{*}\right)$$

$$\begin{matrix} \sum_{j_{A1}\neq j_{B1}}^{J_{i}} \sum_{k_{A1}\neq k_{A2}}^{K_{ij}} \sum_{k_{B1}\neq k_{B2}}^{K_{ij}} (1-\delta_{ij_{A1}k_{B1}})\delta_{ij_{B1}k_{B1}}(1-\delta_{ij_{A1}k_{A2}})\delta_{ij_{B1}k_{B2}}\times\\ COV\left[I(S_{ij_{A1}k_{A1}}-S_{ij_{B1}k_{B1}}),I(S_{ij_{A1}k_{A2}}-S_{ij_{B1}k_{B2}})\right] \end{matrix}$$

$$\left(B_{i1,4}^{*}\right)$$

$$B_{i1,1}^{*}=\sum_{j_{A1}\neq j_{B1}}^{J_{i}} \sum_{k_{A1}=k_{A2}}^{K_{ij}} \sum_{k_{B1}=k_{B2}}^{K_{ij}} (1-\delta_{ij_{A1}k_{A1}})\delta_{ij_{B1}k_{B1}}(1-\delta_{ij_{A1}k_{A1}})\delta_{ij_{B1}k_{B1}}COV\left[I(S_{ij_{A1}k_{A1}}-S_{ij_{B1}k_{B1}}),I(S_{ij_{A1}k_{A1}}-S_{ij_{B1}k_{B1}})\right]=$$

$$B_{i1,2}^{*}=\sum_{j_{A1}\neq j_{B1}}^{J_{i}} \sum_{k_{A1}\neq k_{A2}}^{K_{ij}} \sum_{k_{B1}=k_{B2}}^{K_{ij}} (1-\delta_{ij_{A1}k_{A1}})\delta_{ij_{B1}k_{B1}}(1-\delta_{ij_{A1}k_{A2}})\delta_{ij_{B1}k_{B1}}COV\left[I(S_{ij_{A1}k_{A1}}-S_{ij_{B1}k_{B1}}),I(S_{ij_{A1}k_{A2}}-S_{ij_{B1}k_{B1}})\right]=$$

$$\left[\Phi_{2}\left(\Phi^{-1}(\theta_{f}),\Phi^{-1}(\theta_{f}),\frac{\rho_{s}-2\rho_{f}+1}{2(1-\rho_{f})}\right)-\theta_{f}^{2}\right]\sum_{j_{A1}\neq j_{B1}}^{J_{i}} f_{ij_{B1}}g_{ij_{A1}}(g_{ij_{A1}}-1)$$

$$B_{i1,3}^{*}=\sum_{j_{A1}\neq j_{B1}}^{J_{i}} \sum_{k_{A1}=k_{A2}}^{K_{ij}} \sum_{k_{B1}\neq k_{B2}}^{K_{ij}} (1-\delta_{ij_{A1}k_{A1}})\delta_{ij_{B1}k_{B1}}(1-\delta_{ij_{A1}k_{A1}})\delta_{ij_{B1}k_{B2}}COV\left[I(S_{ij_{A1}k_{A1}}-S_{ij_{B1}k_{B1}}),I(S_{ij_{A1}k_{A1}}-S_{ij_{B1}k_{B2}})\right]=$$

$$\left[\Phi_{2}\left(\Phi^{-1}(\theta_{f}),\Phi^{-1}(\theta_{f}),\frac{\rho_{s}-2\rho_{f}+1}{2(1-\rho_{f})}\right)-\theta_{f}^{2}\right]\sum_{j_{A1}\neq j_{B1}}^{J_{i}} f_{ij_{B1}}(f_{ij_{B1}}-1)g_{ij_{A1}}$$

$$B_{i1,4}^{*}=\sum_{j_{A1}\neq j_{B1}}^{J_{i}} \sum_{k_{A1}\neq k_{A2}}^{K_{ij}} \sum_{k_{B1}\neq k_{B2}}^{K_{ij}} (1-\delta_{ij_{A1}k_{A1}})\delta_{ij_{B1}k_{B1}}(1-\delta_{ij_{A1}k_{A2}})\delta_{ij_{B1}k_{B2}}COV\left[I(S_{ij_{A1}k_{A1}}-S_{ij_{B1}k_{B1}}),I(S_{ij_{A1}k_{A2}}-S_{ij_{B1}k_{B2}})\right]=$$

$$\left[\Phi_{2}\left(\Phi^{-1}(\theta_{f}),\Phi^{-1}(\theta_{f}),\frac{\rho_{s}-\rho_{f}}{1-\rho_{f}}\right)-\theta_{f}^{2}\right]\sum_{j_{A1}\neq j_{B1}}^{J_{i}} g_{ij_{A1}}(g_{ij_{A1}}-1)f_{ij_{B1}}(f_{ij_{B1}}-1)$$

Thus,

(6)

If $\rho_{f}=0$, then (6) reduces to $\Delta B_{1}$ in the Appendix of Rosner, Qiu, and Lee (2013)

$$\begin{matrix} B_{i2}^{*}=\sum_{j_{A1}=j_{A2},j_{A1}\neq j_{B1}}^{J_{i}} \sum_{j_{B1}\neq j_{B2},j_{A2}\neq j_{B2}}^{J_{i}} \sum_{k_{A1}=1}^{K_{ij}} \sum_{k_{A2}=1}^{K_{ij}} \sum_{k_{B1}=1}^{K_{ij}} \sum_{k_{B2}=1}^{K_{ij}} (1-\delta_{ij_{A1}k_{A1}})\delta_{ij_{B1}k_{B1}}(1-\delta_{ij_{A1}k_{A2}})\delta_{ij_{B2}k_{B2}}\times\\ COV\left[I(S_{ij_{A1}k_{A1}}-S_{ij_{B1}k_{B1}}),I(S_{ij_{A1}k_{A2}}-S_{ij_{B2}k_{B2}})\right]= \end{matrix}$$

$$\sum_{j_{A1}\neq j_{B1},j_{A1}\neq j_{B2},j_{B1}\neq j_{B2}}^{J_{i}} \sum_{k_{A1}=k_{A2}}^{K_{ij}} \sum_{k_{B1}=1}^{K_{ij}} \sum_{k_{B2}=1}^{K_{ij}} (1-\delta_{ij_{A1}k_{A1}})\delta_{ij_{B1}k_{B1}}(1-\delta_{ij_{A1}k_{A1}})\delta_{ij_{B2}k_{B2}}\left[I(S_{ij_{A1}k_{A1}}-S_{ij_{B1}k_{B1}}),I(S_{ij_{A1}k_{1}}-S_{ij_{B2}k_{B2}})\right]+$$

$$\sum_{j_{A1}\neq j_{B1},j_{A1}\neq j_{B2},j_{B1}\neq j_{B2}}^{J_{i}} \sum_{k_{A1}\neq k_{A2}}^{K_{ij}} \sum_{k_{B1}=1}^{K_{ij}} \sum_{k_{B2}=1}^{K_{ij}} (1-\delta_{ij_{A1}k_{A1}})\delta_{ij_{B1}k_{B1}}(1-\delta_{ij_{A1}k_{A2}})\delta_{ij_{B2}k_{B2}}\left[I(S_{ij_{A1}k_{A1}}-S_{ij_{B1}k_{B1}}),I(S_{ij_{A1}k_{A2}}-S_{ij_{B2}k_{B2}})\right]$$

$$\begin{matrix} \sum_{k_{A1}=k_{A2}}^{K_{ij}} \sum_{k_{B1}=1}^{K_{ij}} \sum_{k_{B2}=1}^{K_{ij}} (1-\delta_{ij_{A1}k_{A1}})\delta_{ij_{B1}k_{B1}}(1-\delta_{ij_{A1}k_{A1}})\delta_{ij_{B2}k_{B2}}= \\ \sum_{k_{A1}=k_{A2}}^{K_{ij}} (1-\delta_{ij_{A1}k_{A1}})(1-\delta_{ij_{A1}k_{A1}})\sum_{k_{B1}=1}^{K_{ij}} \delta_{ij_{B1}k_{B1}}\sum_{k_{B2}=1}^{K_{ij}} \delta_{ij_{B2}k_{B2}}=g_{j_{A1}}f_{j_{B1}}f_{j_{B2}} \\ \sum_{k_{A1}\neq k_{A2}}^{K_{ij}} \sum_{k_{B1}=1}^{K_{ij}} \sum_{k_{B2}=1}^{K_{ij}} (1-\delta_{ij_{A1}k_{A1}})\delta_{ij_{B1}k_{B1}}(1-\delta_{ij_{A1}k_{A2}})\delta_{ij_{B2}k_{B2}}= \\ \sum_{k_{A1}\neq k_{A2}}^{K_{ij}} (1-\delta_{ij_{A1}k_{A1}})(1-\delta_{ij_{A1}k_{A2}})\sum_{k_{B1}=1}^{K_{ij}} \delta_{ij_{B1}k_{B1}}\sum_{k_{B2}=1}^{K_{ij}} \delta_{ij_{B2}k_{B2}}=g_{j_{A1}}(g_{j_{A1}}-1)f_{j_{B1}}f_{j_{B2}} \end{matrix}$$

$\begin{matrix} B_{i2}^{*}=\left[\Phi_{2}\left(\Phi^{-1}(\theta_{f}),\Phi^{-1}(\theta_{f}),\frac{1}{2}\right)-\theta_{f}^{2}\right]\sum_{j_{A1}\neq j_{B1},j_{A1}\neq j_{B2},j_{B1}\neq j_{B2}}^{J_{i}} g_{j_{A1}}f_{j_{B1}}f_{j_{B2}}+ \\ \left[\Phi_{2}\left(\Phi^{-1}(\theta_{f}),\Phi^{-1}(\theta_{f}),\frac{\rho_{s}-\rho_{f}}{2(1-\rho_{f})}\right)-\theta_{f}^{2}\right]\sum_{j_{A1}\neq j_{B1},j_{A1}\neq j_{B2},j_{B1}\neq j_{B2}}^{J_{i}} g_{j_{A1}}(g_{j_{A1}}-1)f_{j_{B1}}f_{j_{B2}} \end{matrix}$ (7)

If $\rho_{f}=0$, then (7) reduces to $\Delta B_{2}$ in the Appendix of Rosner, Qiu, and Lee (2013)

$$\begin{matrix} B_{i3}^{*}=\sum_{j_{A1}\neq j_{A2},j_{A1}\neq j_{B1}}^{J_{i}} \sum_{j_{B1}=j_{B2},j_{A2}\neq j_{B2}}^{J_{i}} \sum_{k_{A1}=1}^{K_{ij}} \sum_{k_{A2}=1}^{K_{ij}} \sum_{k_{B1}=1}^{K_{ij}} \sum_{k_{B2}=1}^{K_{ij}} (1-\delta_{ij_{A1}k_{A1}})\delta_{ij_{B1}k_{B1}}(1-\delta_{ij_{A2}k_{A2}})\delta_{ij_{B1}k_{B2}}\times\\ COV\left[I(S_{ij_{A1}k_{A1}}-S_{ij_{B1}k_{B1}}),I(S_{ij_{A2}k_{A2}}-S_{ij_{B1}k_{B2}})\right]= \\ \sum_{j_{A1}\neq j_{B1},j_{A1}\neq j_{A2},j_{B1}\neq j_{A2}}^{J_{i}} \sum_{k_{A1}}^{K_{ij}} \sum_{k_{A2}}^{K_{ij}} \sum_{k_{B1}=k_{B2}}^{K_{ij}} (1-\delta_{ij_{A1}k_{A1}})\delta_{ij_{B1}k_{B1}}(1-\delta_{ij_{A2}k_{A2}})\delta_{ij_{B1}k_{B1}}\times\\ \left[I(S_{ij_{A1}k_{A1}}-S_{ij_{B1}k_{B1}}),I(S_{ij_{A2}k_{A2}}-S_{ij_{B1}k_{B1}})\right]+ \\ \sum_{j_{A1}\neq j_{B1},j_{A1}\neq j_{A2},j_{B1}\neq j_{A2}}^{J_{i}} \sum_{k_{A1}}^{K_{ij}} \sum_{k_{A2}}^{K_{ij}} \sum_{k_{B1}\neq k_{B2}}^{K_{ij}} (1-\delta_{ij_{A1}k_{A1}})\delta_{ij_{B1}k_{B1}}(1-\delta_{ij_{A2}k_{A2}})\delta_{ij_{B1}k_{B2}}\times\\ \left[I(S_{ij_{A1}k_{A1}}-S_{ij_{B1}k_{B1}}),I(S_{ij_{A2}k_{A2}}-S_{ij_{B1}k_{B2}})\right] \end{matrix}$$

$$\begin{matrix} \sum_{k_{A1}}^{K_{ij}} \sum_{k_{A2}}^{K_{ij}} \sum_{k_{B1}=k_{B2}}^{K_{ij}} (1-\delta_{ij_{A1}k_{A1}})\delta_{ij_{B1}k_{B1}}(1-\delta_{ij_{A2}k_{A2}})\delta_{ij_{B1}k_{B1}}= \\ \sum_{k_{A1}}^{K_{ij}} (1-\delta_{ij_{A1}k_{A1}})\sum_{k_{A2}}^{K_{ij}} (1-\delta_{ij_{A2}k_{A2}})\sum_{k_{B1}=k_{B2}}^{K_{ij}} \delta_{ij_{B1}k_{B1}}=g_{j_{A1}}g_{j_{A2}}f_{j_{B1}} \\ \sum_{k_{A1}}^{K_{ij}} \sum_{k_{A2}}^{K_{ij}} \sum_{k_{B1}\neq k_{B2}}^{K_{ij}} (1-\delta_{ij_{A1}k_{A1}})\delta_{ij_{B1}k_{B1}}(1-\delta_{ij_{A2}k_{A2}})\delta_{ij_{B1}k_{B2}}= \\ \sum_{k_{A1}}^{K_{ij}} (1-\delta_{ij_{A1}k_{A1}})\sum_{k_{A2}}^{K_{ij}} (1-\delta_{ij_{A2}k_{A2}})\sum_{k_{B1}\neq k_{B2}}^{K_{ij}} \delta_{ij_{B1}k_{B1}}\delta_{ij_{B1}k_{B2}}=g_{j_{A1}}g_{j_{A2}}f_{j_{B1}}(f_{j_{B1}}-1) \end{matrix}$$

$\begin{matrix} B_{i3}^{*}=\left[\Phi_{2}\left(\Phi^{-1}(\theta_{f}),\Phi^{-1}(\theta_{f}),\frac{1}{2}\right)-\theta_{f}^{2}\right]\sum_{j_{A1}\neq j_{B1},j_{A1}\neq j_{A2},j_{B1}\neq j_{A2}}^{J_{i}} g_{j_{A1}}g_{j_{A2}}f_{j_{B1}}+ \\ \left[\Phi_{2}\left(\Phi^{-1}(\theta_{f}),\Phi^{-1}(\theta_{f}),\frac{\rho_{s}-\rho_{f}}{2(1-\rho_{f})}\right)-\theta_{f}^{2}\right]\sum_{j_{A1}\neq j_{B1},j_{A1}\neq j_{A2},j_{B1}\neq j_{A2}}^{J_{i}} g_{j_{A1}}g_{j_{A2}}f_{j_{B1}}(f_{j_{B1}}-1) \end{matrix}$ (8)

If $\rho_{f}=0$, then (8) reduces to $\Delta B_{3}$ in the Appendix of Rosner, Qiu, and Lee (2013)

$$\begin{matrix} B_{i4}^{*}=\sum_{j_{A1}=j_{B2},j_{A1}\neq j_{B1}}^{J_{i}} \sum_{j_{B1}\neq j_{A2},j_{A2}\neq j_{B2}}^{J_{i}} \sum_{k_{A1}=1}^{K_{ij}} \sum_{k_{A2}=1}^{K_{ij}} \sum_{k_{B1}=1}^{K_{ij}} \sum_{k_{B2}=1}^{K_{ij}} (1-\delta_{ij_{A1}k_{A1}})\delta_{ij_{B1}k_{B1}}(1-\delta_{ij_{B1}k_{A2}})\delta_{ij_{A1}k_{B2}}\times\\ COV\left[I(S_{ij_{A1}k_{A1}}-S_{ij_{B1}k_{B1}}),I(S_{ij_{B1}k_{A2}}-S_{ij_{A1}k_{B2}})\right]= \\ \sum_{j_{A1}\neq j_{B1}}^{J_{i}} \sum_{k_{A1}=1}^{K_{ij}} \sum_{k_{A2}=1}^{K_{ij}} \sum_{k_{B1}=1}^{K_{ij}} \sum_{k_{B2}=1}^{K_{ij}} (1-\delta_{ij_{A1}k_{A1}})\delta_{ij_{B1}k_{B1}}(1-\delta_{ij_{B1}k_{A2}})\delta_{ij_{A1}k_{B2}}\times\\ COV\left[I(S_{ij_{A1}k_{A1}}-S_{ij_{B1}k_{B1}}),I(S_{ij_{B1}k_{A2}}-S_{ij_{A1}k_{B2}})\right] \end{matrix}$$

$$\begin{matrix} \sum_{k_{A1}=1}^{K_{ij}} \sum_{k_{A2}=1}^{K_{ij}} \sum_{k_{B1}=1}^{K_{ij}} \sum_{k_{B2}=1}^{K_{ij}} (1-\delta_{ij_{A1}k_{A1}})\delta_{ij_{B1}k_{B1}}(1-\delta_{ij_{B1}k_{A2}})\delta_{ij_{A1}k_{B2}}= \\ \sum_{k_{A1}=1}^{K_{ij}} (1-\delta_{ij_{A1}k_{A1}})\sum_{k_{A2}=1}^{K_{ij}} (1-\delta_{ij_{B1}k_{A2}})\sum_{k_{B1}=1}^{K_{ij}} \delta_{ij_{B1}k_{B1}}\sum_{k_{B2}=1}^{K_{ij}} \delta_{ij_{A1}k_{B2}}=g_{j_{A1}}g_{j_{B1}}f_{j_{B1}}f_{j_{A1}} \end{matrix}$$

$B_{i4}^{*}=\left[\Phi_{2}\left(\Phi^{-1}(\theta_{f}),\Phi^{-1}(\theta_{f}),\frac{\rho_{f}-\rho_{s}}{(1-\rho_{f})}\right)-\theta_{f}^{2}\right]\sum_{j_{A1}\neq j_{B1}}^{J_{i}} f_{j_{A1}}f_{j_{B1}}g_{j_{B1}}g_{j_{A1}}$ (9)

If $\rho_{f}=0$, then (9) reduces to $\Delta B_{4}$ in the Appendix of Rosner, Qiu, and Lee (2013)

$$\begin{matrix} B_{i5}^{*}=2\times\sum_{j_{A1}=j_{B2},j_{A1}\neq j_{B1}}^{J_{i}} \sum_{j_{B1}\neq j_{A2},j_{A2}\neq j_{B2}}^{J_{i}} \sum_{k_{A1}=1}^{K_{ij}} \sum_{k_{A2}=1}^{K_{ij}} \sum_{k_{B1}=1}^{K_{ij}} \sum_{k_{B2}=1}^{K_{ij}} (1-\delta_{ij_{A1}k_{A1}})\delta_{ij_{B1}k_{B1}}(1-\delta_{ij_{A2}k_{A2}})\delta_{ij_{A1}k_{B2}}\times\\ COV\left[I(S_{ij_{A1}k_{A1}}-S_{ij_{B1}k_{B1}}),I(S_{ij_{A2}k_{A2}}-S_{ij_{A1}k_{B2}})\right]= \\ 2\times\sum_{j_{A1}\neq j_{B1},j_{A1}\neq j_{A2},j_{B1}\neq j_{A2}}^{J_{i}} \sum_{k_{A1}=1}^{K_{ij}} \sum_{k_{A2}=1}^{K_{ij}} \sum_{k_{B1}=1}^{K_{ij}} \sum_{k_{B2}=1}^{K_{ij}} (1-\delta_{ij_{A1}k_{A1}})\delta_{ij_{B1}k_{B1}}(1-\delta_{ij_{A2}k_{A2}})\delta_{ij_{A1}k_{B2}}\times\\ COV\left[I(S_{ij_{A1}k_{A1}}-S_{ij_{B1}k_{B1}}),I(S_{ij_{A2}k_{A2}}-S_{ij_{A1}k_{B2}})\right] \end{matrix}$$

$$\begin{matrix} \sum_{k_{A1}=1}^{K_{ij}} \sum_{k_{A2}=1}^{K_{ij}} \sum_{k_{B1}=1}^{K_{ij}} \sum_{k_{B2}=1}^{K_{ij}} (1-\delta_{ij_{A1}k_{A1}})\delta_{ij_{B1}k_{B1}}(1-\delta_{ij_{A2}k_{A2}})\delta_{ij_{A1}k_{B2}}= \\ \sum_{k_{A1}=1}^{K_{ij}} (1-\delta_{ij_{A1}k_{A1}})\sum_{k_{A2}=1}^{K_{ij}} (1-\delta_{ij_{A2}k_{A2}})\sum_{k_{B1}=1}^{K_{ij}} \delta_{ij_{B1}k_{B1}}\sum_{k_{B2}=1}^{K_{ij}} \delta_{ij_{A1}k_{B2}}=g_{j_{A1}}g_{j_{A2}}f_{j_{B1}}f_{j_{A1}} \end{matrix}$$

$B_{i5}^{*}=2\times\left[\Phi_{2}\left(\Phi^{-1}(\theta_{f}),\Phi^{-1}(\theta_{f}),\frac{\rho_{f}-\rho_{s}}{2(1-\rho_{f})}\right)-\theta_{f}^{2}\right]\sum_{j_{A1}\neq j_{B1},j_{A1}\neq j_{A2},j_{B1}\neq j_{A2}}^{J_{i}} g_{j_{A1}}g_{j_{A2}}f_{j_{B1}}f_{j_{A1}}$ (10)

If $\rho_{f}=0$, then (10) reduces to $\Delta B_{5}$ in the Appendix of Rosner, Qiu, and Lee (2013)

Thus,

$$VAR(B)=\sum_{i=1}^{I} VAR(B_{i})=\sum_{i=1}^{I} \sum_{y=1}^{5} B_{iy}^{*}$$

4. *VAR(C)*

$$C=\sum_{i_{1}=1}^{I} \sum_{i_{2}\neq i_{1}}^{I} \sum_{j_{1}=1}^{J_{i_{1}}} \sum_{j_{2}=1}^{J_{i_{2}}} \sum_{k_{1}=1}^{K_{i_{1}j_{1}}} \sum_{k_{2}=1}^{K_{i_{2}j_{2}}} (1-\delta_{i_{1}j_{1}k_{1}})\delta_{i_{2}j_{2}k_{2}}I(S_{i_{1}j_{1}k_{1}}-S_{i_{2}j_{2}k_{2}})$$

$$VAR(C)=VAR\left[\sum_{i_{1}=1}^{I} \sum_{i_{2}\neq i_{1}}^{I} \sum_{j_{1}=1}^{J_{i_{1}}} \sum_{j_{2}=1}^{J_{i_{2}}} \sum_{k_{1}=1}^{K_{i_{1}j_{1}}} \sum_{k_{2}=1}^{K_{i_{2}j_{2}}} (1-\delta_{i_{1}j_{1}k_{1}})\delta_{i_{2}j_{2}k_{2}}I(S_{i_{1}j_{1}k_{1}}-S_{i_{2}j_{2}k_{2}})\right]=$$

$\begin{matrix} \sum_{i_{A1}}^{I} \sum_{i_{B1}}^{I} \sum_{i_{A2}}^{I} \sum_{i_{B2}}^{I} \sum_{j_{A1}}^{J_{i_{A1}}} \sum_{j_{B1}}^{J_{i_{B1}}} \sum_{j_{A2}}^{J_{i_{A2}}} \sum_{j_{B2}}^{J_{i_{B2}}} \sum_{k_{A1}}^{K_{i_{A1}j_{A1}}} \sum_{k_{B1}}^{K_{i_{B1}j_{B1}}} \sum_{k_{A2}}^{K_{i_{A2}j_{A2}}} \sum_{k_{B2}}^{K_{i_{B2}j_{B2}}} (1-\delta_{i_{A1}j_{A1}k_{A1}})\delta_{i_{B1}j_{B1}k_{B1}}\times\\ (1-\delta_{i_{A2}j_{A2}k_{A2}})\delta_{i_{B2}j_{B2}k_{B2}}COV\left[I(S_{i_{A1}j_{A1}k_{A1}}-S_{i_{B1}j_{B1}k_{B1}}),I(S_{i_{A2}j_{A2}k_{A2}}-S_{i_{B2}j_{B2}k_{B2}})\right] \end{matrix}$ (11)

There are six different patterns of family-level similarity that can occur when taking the covariance of two score differences from members of different families (*W≠X≠Y≠Z*).

| *WX* | *YZ* | 0 |
| --- | --- | --- |
| *WX* | *WX* | $C_{1}^{*}$ |
| *WX* | *YW* | $C_{2}^{*}$ |
| *WX* | *WY* | $C_{3}^{*}$ |
| *WX* | *YX* | $C_{4}^{*}$ |
| *WX* | *XY* | $C_{5}^{*}$ |
| *WX* | *XW* | $C_{6}^{*}$ |

$$\begin{matrix} C_{1}^{*}=\sum_{i_{A1},i_{A2}=i_{A1}}^{I} \sum_{i_{B1}\neq i_{A1},i_{B2}=i_{B1}}^{I} \sum_{j_{A1}}^{J_{i_{A1}}} \sum_{j_{B1}}^{J_{i_{B1}}} \sum_{j_{A2}}^{J_{i_{A2}}} \sum_{j_{B2}}^{J_{i_{B2}}} \sum_{k_{A1}}^{K_{i_{A1}j_{A1}}} \sum_{k_{B1}}^{K_{i_{B1}j_{B1}}} \sum_{k_{A2}}^{K_{i_{A2}j_{A2}}} \sum_{k_{B2}}^{K_{i_{B2}j_{B2}}} (1-\delta_{i_{A1}j_{A1}k_{A1}})\delta_{i_{B1}j_{B1}k_{B1}}\times\\ (1-\delta_{i_{A1}j_{A2}k_{A2}})\delta_{i_{B1}j_{B2}k_{B2}}COV\left[I(S_{i_{A1}j_{A1}k_{A1}}-S_{i_{B1}j_{B1}k_{B1}}),I(S_{i_{A1}j_{A2}k_{A2}}-S_{i_{B1}j_{B2}k_{B2}})\right]= \end{matrix}$$

$$\begin{matrix} \sum_{i_{A1}=i_{A2},i_{B1}=i_{B2}}^{I} \sum_{j_{A1}}^{J_{i_{A1}}} \sum_{j_{B1}}^{J_{i_{B1}}} \sum_{j_{A2}\neq j_{A1}}^{J_{i_{A2}}} \sum_{j_{B2}\neq j_{B1}}^{J_{i_{B2}}} \sum_{k_{A1}}^{K_{i_{A1}j_{A1}}} \sum_{k_{B1}}^{K_{i_{B1}j_{B1}}} \sum_{k_{A2}}^{K_{i_{A2}j_{A2}}} \sum_{k_{B2}}^{K_{i_{B2}j_{B2}}} (1-\delta_{i_{A1}j_{A1}k_{A1}})\times\\ \delta_{i_{B1}j_{B1}k_{B1}}(1-\delta_{i_{A1}j_{A2}k_{A2}})\delta_{i_{B1}j_{B2}k_{B2}}\times\\ COV\left[I(S_{i_{A1}j_{A1}k_{A1}}-S_{i_{B1}j_{B1}k_{B1}}),I(S_{i_{A1}j_{A2}k_{A2}}-S_{i_{B1}j_{B2}k_{B2}})\right]+ \end{matrix}$$

$$(C_{1,1}^{*})$$

$$\begin{matrix} \sum_{i_{A1}=i_{A2},i_{B1}=i_{B2}}^{I} \sum_{j_{A1}}^{J_{i_{A1}}} \sum_{j_{B1},j_{B2}=j_{B1}}^{J_{i_{B1}}} \sum_{j_{A2}\neq j_{A1}}^{J_{i_{A2}}} \sum_{k_{A1}}^{K_{i_{A1}j_{A1}}} \sum_{k_{B1}}^{K_{i_{B1}j_{B1}}} \sum_{k_{A2}}^{K_{i_{A2}j_{A2}}} \sum_{k_{B2}\neq k_{B1}}^{K_{i_{B2}j_{B2}}} (1-\delta_{i_{A1}j_{A1}k_{A1}})\times\\ \delta_{i_{B1}j_{B1}k_{B1}}(1-\delta_{i_{A1}j_{A2}k_{A2}})\delta_{i_{B1}j_{B1}k_{B2}}\times\\ COV\left[I(S_{i_{A1}j_{A1}k_{A1}}-S_{i_{B1}j_{B1}k_{B1}}),I(S_{i_{A1}j_{A2}k_{A2}}-S_{i_{B1}j_{B1}k_{B2}})\right]+ \end{matrix}$$

$$(C_{1,2}^{*})$$

$$\begin{matrix} \sum_{i_{A1}=i_{A2},i_{B1}=i_{B2}}^{I} \sum_{j_{A1}}^{J_{i_{A1}}} \sum_{j_{B1},j_{B2}=j_{B1}}^{J_{i_{B1}}} \sum_{j_{A2}\neq j_{A1}}^{J_{i_{A2}}} \sum_{k_{A1}}^{K_{i_{A1}j_{A1}}} \sum_{k_{B1},k_{B2}=k_{B1}}^{K_{i_{B1}j_{B1}}} \sum_{k_{A2}}^{K_{i_{A2}j_{A2}}} (1-\delta_{i_{A1}j_{A1}k_{A1}})\times\\ \delta_{i_{B1}j_{B1}k_{B1}}(1-\delta_{i_{A1}j_{A2}k_{A2}})\delta_{i_{B1}j_{B1}k_{B1}}\times\\ COV\left[I(S_{i_{A1}j_{A1}k_{A1}}-S_{i_{B1}j_{B1}k_{B1}}),I(S_{i_{A1}j_{A2}k_{A2}}-S_{i_{B1}j_{B1}k_{B1}})\right]+ \end{matrix}$$

$$(C_{1,3}^{*})$$

$$\begin{matrix} \sum_{i_{A1}=i_{A2},i_{B1}=i_{B2}}^{I} \sum_{j_{A1},j_{A2}=j_{A1}}^{J_{i_{A1}}} \sum_{j_{B1}}^{J_{i_{B1}}} \sum_{j_{B2}\neq j_{B1}}^{J_{i_{B2}}} \sum_{k_{A1}}^{K_{i_{A1}j_{A1}}} \sum_{k_{B1}}^{K_{i_{B1}j_{B1}}} \sum_{k_{A2}\neq k_{A1}}^{K_{i_{A2}j_{A2}}} \sum_{k_{B2}}^{K_{i_{B2}j_{B2}}} (1-\delta_{i_{A1}j_{A1}k_{A1}})\times\\ \delta_{i_{B1}j_{B1}k_{B1}}(1-\delta_{i_{A1}j_{A1}k_{A2}})\delta_{i_{B1}j_{B2}k_{B2}}\times\\ COV\left[I(S_{i_{A1}j_{A1}k_{A1}}-S_{i_{B1}j_{B1}k_{B1}}),I(S_{i_{A1}j_{A1}k_{A2}}-S_{i_{B1}j_{B2}k_{B2}})\right]+ \end{matrix}$$

$$(C_{1,4}^{*})$$

$$\begin{matrix} \sum_{i_{A1}=i_{A2},i_{B1}=i_{B2}}^{I} \sum_{j_{A1},j_{A2}=j_{A1}}^{J_{i_{A1}}} \sum_{j_{B1}}^{J_{i_{B1}}} \sum_{j_{B2}\neq j_{B1}}^{J_{i_{B2}}} \sum_{k_{A1},k_{A2}=k_{A1}}^{K_{i_{A1}j_{A1}}} \sum_{k_{B1}}^{K_{i_{B1}j_{B1}}} \sum_{k_{B2}}^{K_{i_{B2}j_{B2}}} (1-\delta_{i_{A1}j_{A1}k_{A1}})\times\\ \delta_{i_{B1}j_{B1}k_{B1}}(1-\delta_{i_{A1}j_{A1}k_{A1}})\delta_{i_{B1}j_{B2}k_{B2}}\times\\ COV\left[I(S_{i_{A1}j_{A1}k_{A1}}-S_{i_{B1}j_{B1}k_{B1}}),I(S_{i_{A1}j_{A1}k_{A1}}-S_{i_{B1}j_{B2}k_{B2}})\right]+ \end{matrix}$$

$$(C_{1,5}^{*})$$

$$\begin{matrix} \sum_{i_{A1}=i_{A2},i_{B1}=i_{B2}}^{I} \sum_{j_{A1},j_{A2}=j_{A1}}^{J_{i_{A1}}} \sum_{j_{B1},j_{B2}=j_{B1}}^{J_{i_{B1}}} \sum_{k_{A1}}^{K_{i_{A1}j_{A1}}} \sum_{k_{B1}}^{K_{i_{B1}j_{B1}}} \sum_{k_{A2}\neq k_{A1}}^{K_{i_{A2}j_{A2}}} \sum_{k_{B2}\neq k_{B1}}^{K_{i_{B2}j_{B2}}} (1-\delta_{i_{A1}j_{A1}k_{A1}})\times\\ \delta_{i_{B1}j_{B1}k_{B1}}(1-\delta_{i_{A1}j_{A1}k_{A2}})\delta_{i_{B1}j_{B1}k_{B2}}\times\\ COV\left[I(S_{i_{A1}j_{A1}k_{A1}}-S_{i_{B1}j_{B1}k_{B1}}),I(S_{i_{A1}j_{A1}k_{A2}}-S_{i_{B1}j_{B1}k_{B2}})\right]+ \end{matrix}$$

$$(C_{1,6}^{*})$$

$$\begin{matrix} \sum_{i_{A1}=i_{A2},i_{B1}=i_{B2}}^{I} \sum_{j_{A1},j_{A2}=j_{A1}}^{J_{i_{A1}}} \sum_{j_{B1},j_{B2}=j_{B1}}^{J_{i_{B1}}} \sum_{k_{A1}}^{K_{i_{A1}j_{A1}}} \sum_{k_{B1},k_{B2}=k_{B1}}^{K_{i_{B1}j_{B1}}} \sum_{k_{A2}\neq k_{A1}}^{K_{i_{A2}j_{A2}}} (1-\delta_{i_{A1}j_{A1}k_{A1}})\times\\ \delta_{i_{B1}j_{B1}k_{B1}}(1-\delta_{i_{A1}j_{A1}k_{A2}})\delta_{i_{B1}j_{B1}k_{B1}}\times\\ COV\left[I(S_{i_{A1}j_{A1}k_{A1}}-S_{i_{B1}j_{B1}k_{B1}}),I(S_{i_{A1}j_{A1}k_{A2}}-S_{i_{B1}j_{B1}k_{B1}})\right]+ \end{matrix}$$

$$(C_{1,7}^{*})$$

$$\begin{matrix} \sum_{i_{A1}=i_{A2},i_{B1}=i_{B2}}^{I} \sum_{j_{A1},j_{A2}=j_{A1}}^{J_{i_{A1}}} \sum_{j_{B1},j_{B2}=j_{B1}}^{J_{i_{B1}}} \sum_{k_{A1},k_{A2}=k_{A1}}^{K_{i_{A1}j_{A1}}} \sum_{k_{B1}}^{K_{i_{B1}j_{B1}}} \sum_{k_{B2}\neq k_{B1}}^{K_{i_{B2}j_{B2}}} (1-\delta_{i_{A1}j_{A1}k_{A1}})\times\\ \delta_{i_{B1}j_{B1}k_{B1}}(1-\delta_{i_{A1}j_{A1}k_{A1}})\delta_{i_{B1}j_{B1}k_{B2}}\times\\ COV\left(I(S_{i_{A1}j_{A1}k_{A1}}-S_{i_{B1}j_{B1}k_{B1}}),I(S_{i_{A1}j_{A1}k_{A1}}-S_{i_{B1}j_{B1}k_{B2}})\right)+ \end{matrix}$$

$$(C_{1,8}^{*})$$

$$\begin{matrix} \sum_{i_{A1}=i_{A2},i_{B1}=i_{B2}}^{I} \sum_{j_{A1},j_{A2}=j_{A1}}^{J_{i_{A1}}} \sum_{j_{B1},j_{B2}=j_{B1}}^{J_{i_{B1}}} \sum_{k_{A1},k_{A2}=k_{A1}}^{K_{i_{A1}j_{A1}}} \sum_{k_{B1},k_{B2}=k_{B1}}^{K_{i_{B1}j_{B1}}} (1-\delta_{i_{A1}j_{A1}k_{A1}})\delta_{i_{B1}j_{B1}k_{B1}}\times\\ VAR\left[I(S_{i_{A1}j_{A1}k_{A1}}-S_{i_{B1}j_{B1}k_{B1}})\right] \end{matrix}$$

$$(C_{1,9}^{*})$$

$$\begin{matrix} C_{1,1}^{*}=\left[\Phi_{2}\left(\Phi^{-1}(\theta),\Phi^{-1}(\theta),\rho_{f}\right)-\theta^{2}\right]\sum_{i_{A1}=i_{A2},i_{B1}=i_{B2}}^{I} \sum_{j_{A1}}^{J_{i_{A1}}} \sum_{j_{B1}}^{J_{i_{B1}}} \sum_{j_{A2}\neq j_{A1}}^{J_{i_{A2}}} \sum_{j_{B2}\neq j_{B1}}^{J_{i_{B2}}} \sum_{k_{A1}}^{K_{i_{A1}j_{A1}}} (1-\delta_{i_{A1}j_{A1}k_{A1}})\times\\ \sum_{k_{B1}}^{K_{i_{B1}j_{B1}}} \delta_{i_{B1}j_{B1}k_{B1}}\sum_{k_{A2}}^{K_{i_{A2}j_{A2}}} (1-\delta_{i_{A1}j_{A2}k_{A2}})\sum_{k_{B2}}^{K_{i_{B2}j_{B2}}} \delta_{i_{B1}j_{B2}k_{B2}}= \\ \left[\Phi_{2}\left(\Phi^{-1}(\theta),\Phi^{-1}(\theta),\rho_{f}\right)-\theta^{2}\right]\sum_{i_{A1}=i_{A2},i_{B1}=i_{B2}}^{I} \sum_{j_{A1}}^{J_{i_{A1}}} \sum_{j_{A2}\neq j_{A1}}^{J_{i_{A2}}} g_{i_{A1}j_{A1}}g_{i_{A1}j_{A2}}\sum_{j_{B1}}^{J_{i_{B1}}} \sum_{j_{B2}\neq j_{B1}}^{J_{i_{B2}}} f_{i_{B1}j_{B1}}f_{i_{B1}j_{B2}}= \\ \left[\Phi_{2}\left(\Phi^{-1}(\theta),\Phi^{-1}(\theta),\rho_{f}\right)-\theta^{2}\right]\sum_{i_{A1}=i_{A2},i_{B1}=i_{B2}}^{I} \left(g_{i_{A2}}^{2}-\sum_{j_{A1}}^{J_{i_{A1}}} g_{i_{A1}j_{A1}}^{2}\right)\left(f_{i_{B2}}^{2}-\sum_{j_{B1}}^{J_{i_{B1}}} f_{i_{B1}j_{B1}}^{2}\right) \end{matrix}$$

$$\begin{matrix} C_{1,2}^{*}=\left[\Phi_{2}\left(\Phi^{-1}(\theta),\Phi^{-1}(\theta),\frac{\rho_{f}+\rho_{s}}{2}\right)-\theta^{2}\right]\times\\ \sum_{i_{A1}=i_{A2},i_{B1}=i_{B2}}^{I} \sum_{j_{A1}}^{J_{i_{A1}}} \sum_{j_{B1},j_{B2}=j_{B1}}^{J_{i_{B1}}} \sum_{j_{A2}\neq j_{A1}}^{J_{i_{A2}}} \sum_{k_{A1}}^{K_{i_{A1}j_{A1}}} (1-\delta_{i_{A1}j_{A1}k_{A1}})\sum_{k_{B1}}^{K_{i_{B1}j_{B1}}} \delta_{i_{B1}j_{B1}k_{B1}}\sum_{k_{A2}}^{K_{i_{A2}j_{A2}}} (1-\delta_{i_{A1}j_{A2}k_{A2}})\sum_{k_{B2}\neq k_{B1}}^{K_{i_{B2}j_{B2}}} \delta_{i_{B1}j_{B1}k_{B2}} \end{matrix}$$

$$\begin{matrix} C_{1,3}^{*}=\left[\Phi_{2}\left(\Phi^{-1}(\theta),\Phi^{-1}(\theta),\frac{\rho_{f}+1}{2}\right)-\theta^{2}\right]\times\\ \sum_{i_{A1}=i_{A2},i_{B1}=i_{B2}}^{I} \sum_{j_{A1}}^{J_{i_{A1}}} \sum_{j_{B1},j_{B2}=j_{B1}}^{J_{i_{B1}}} \sum_{j_{A2}\neq j_{A1}}^{J_{i_{A2}}} \sum_{k_{A1}}^{K_{i_{A1}j_{A1}}} (1-\delta_{i_{A1}j_{A1}k_{A1}})\sum_{k_{B1},k_{B2}=k_{B1}}^{K_{i_{B1}j_{B1}}} \delta_{i_{B1}j_{B1}k_{B1}}\delta_{i_{B1}j_{B1}k_{B1}}\sum_{k_{A2}}^{K_{i_{A2}j_{A2}}} (1-\delta_{i_{A1}j_{A2}k_{A2}}) \end{matrix}$$

$$\begin{matrix} C_{1,4}^{*}=\left[\Phi_{2}\left(\Phi^{-1}(\theta),\Phi^{-1}(\theta),\frac{\rho_{s}+\rho_{f}}{2}\right)-\theta^{2}\right]\times\\ \sum_{i_{A1}=i_{A2},i_{B1}=i_{B2}}^{I} \sum_{j_{A1}}^{J_{i_{A1}}} \sum_{j_{B1}}^{J_{i_{B1}}} \sum_{j_{A2}=j_{A1}}^{J_{i_{A2}}} \sum_{j_{B2}\neq j_{B1}}^{J_{i_{B2}}} \sum_{k_{A1}}^{K_{i_{A1}j_{A1}}} (1-\delta_{i_{A1}j_{A1}k_{A1}})\sum_{k_{B1}}^{K_{i_{B1}j_{B1}}} \delta_{i_{B1}j_{B1}k_{B1}}\sum_{k_{A2}\neq k_{A1}}^{K_{i_{A2}j_{A2}}} (1-\delta_{i_{A1}j_{A1}k_{A2}})\sum_{k_{B2}}^{K_{i_{B2}j_{B2}}} \delta_{i_{B1}j_{B2}k_{B2}} \end{matrix}$$

$$\begin{matrix} C_{1,5}^{*}=\left[\Phi_{2}\left(\Phi^{-1}(\theta),\Phi^{-1}(\theta),\frac{1+\rho_{f}}{2}\right)-\theta^{2}\right]\times\\ \sum_{i_{A1}=i_{A2},i_{B1}=i_{B2}}^{I} \sum_{j_{A1}}^{J_{i_{A1}}} \sum_{j_{B1}}^{J_{i_{B1}}} \sum_{j_{A2}=j_{A1}}^{J_{i_{A2}}} \sum_{j_{B2}\neq j_{B1}}^{J_{i_{B2}}} \sum_{k_{A1}}^{K_{i_{A1}j_{A1}}} (1-\delta_{i_{A1}j_{A1}k_{A1}})\sum_{k_{B1}}^{K_{i_{B1}j_{B1}}} \delta_{i_{B1}j_{B1}k_{B1}}\sum_{k_{A2}=k_{A1}}^{K_{i_{A2}j_{A2}}} (1-\delta_{i_{A1}j_{A1}k_{A1}})\sum_{k_{B2}}^{K_{i_{B2}j_{B2}}} \delta_{i_{B1}j_{B2}k_{B2}} \end{matrix}$$

$$\begin{matrix} C_{1,6}^{*}=\left[\Phi_{2}\left(\Phi^{-1}(\theta),\Phi^{-1}(\theta),\rho_{s}\right)-\theta^{2}\right]\sum_{i_{A1}=i_{A2},i_{B1}=i_{B2}}^{I} \sum_{j_{A1}}^{J_{i_{A1}}} \sum_{j_{B1}}^{J_{i_{B1}}} \sum_{j_{A2}=j_{A1}}^{J_{i_{A2}}} \sum_{j_{B2}=j_{B1}}^{J_{i_{B2}}} \sum_{k_{A1}}^{K_{i_{A1}j_{A1}}} (1-\delta_{i_{A1}j_{A1}k_{A1}})\times\\ \sum_{k_{B1}}^{K_{i_{B1}j_{B1}}} \delta_{i_{B1}j_{B1}k_{B1}}\sum_{k_{A2}\neq k_{A1}}^{K_{i_{A2}j_{A2}}} (1-\delta_{i_{A1}j_{A1}k_{A2}})\sum_{k_{B2}\neq k_{B1}}^{K_{i_{B2}j_{B2}}} \delta_{i_{B1}j_{B1}k_{B2}} \end{matrix}$$

$$\begin{matrix} C_{1,7}^{*}=\left[\Phi_{2}\left(\Phi^{-1}(\theta),\Phi^{-1}(\theta),\frac{\rho_{s}+1}{2}\right)-\theta^{2}\right]\sum_{i_{A1}=i_{A2},i_{B1}=i_{B2}}^{I} \sum_{j_{A1}}^{J_{i_{A1}}} \sum_{j_{B1}}^{J_{i_{B1}}} \sum_{j_{A2}=j_{A1}}^{J_{i_{A2}}} \sum_{j_{B2}=j_{B1}}^{J_{i_{B2}}} \sum_{k_{A1}}^{K_{i_{A1}j_{A1}}} (1-\delta_{i_{A1}j_{A1}k_{A1}})\times\\ \sum_{k_{B1}}^{K_{i_{B1}j_{B1}}} \delta_{i_{B1}j_{B1}k_{B1}}\sum_{k_{A2}\neq k_{A1}}^{K_{i_{A2}j_{A2}}} (1-\delta_{i_{A1}j_{A1}k_{A2}})\sum_{k_{B2}=k_{B1}}^{K_{i_{B2}j_{B2}}} \delta_{i_{B1}j_{B1}k_{B1}} \end{matrix}$$

$$\begin{matrix} C_{1,8}^{*}=\left[\Phi_{2}\left(\Phi^{-1}(\theta),\Phi^{-1}(\theta),\frac{1+\rho_{s}}{2}\right)-\theta^{2}\right]\sum_{i_{A1}=i_{A2},i_{B1}=i_{B2}}^{I} \sum_{j_{A1}}^{J_{i_{A1}}} \sum_{j_{B1}}^{J_{i_{B1}}} \sum_{j_{A2}=j_{A1}}^{J_{i_{A2}}} \sum_{j_{B2}=j_{B1}}^{J_{i_{B2}}} \sum_{k_{A1}}^{K_{i_{A1}j_{A1}}} (1-\delta_{i_{A1}j_{A1}k_{A1}})\times\\ \sum_{k_{B1}}^{K_{i_{B1}j_{B1}}} \delta_{i_{B1}j_{B1}k_{B1}}\sum_{k_{A2}=k_{A1}}^{K_{i_{A2}j_{A2}}} (1-\delta_{i_{A1}j_{A1}k_{A1}})\sum_{k_{B2}\neq k_{B1}}^{K_{i_{B2}j_{B2}}} \delta_{i_{B1}j_{B1}k_{B2}} \end{matrix}$$

$$C_{1,9}^{*}=\theta(1-\theta)\sum_{i_{A1}=i_{A2},i_{B1}=i_{B2}}^{I} \sum_{j_{A1}}^{J_{i_{A1}}} \sum_{j_{B1}}^{J_{i_{B1}}} \sum_{j_{A2}=j_{A1}}^{J_{i_{A2}}} \sum_{j_{B2}=j_{B1}}^{J_{i_{B2}}} \sum_{k_{A1}}^{K_{i_{A1}j_{A1}}} \sum_{k_{B1}}^{K_{i_{B1}j_{B1}}} \sum_{k_{A2}=k_{A1}}^{K_{i_{A2}j_{A2}}} \sum_{k_{B2}=k_{B1}}^{K_{i_{B2}j_{B2}}} (1-\delta_{i_{A1}j_{A1}k_{A1}})\delta_{i_{B1}j_{B1}k_{B1}}$$

$C_{1}^{*}=C_{1,1}^{*}+C_{1,2}^{*}+C_{1,3}^{*}+C_{1,4}^{*}+C_{1,5}^{*}+C_{1,6}^{*}+C_{1,7}^{*}+C_{1,8}^{*}+C_{1,9}^{*}$ (12)

$$\begin{matrix} C_{2}^{*}=\sum_{i_{A1}}^{I} \sum_{i_{B1}\neq i_{A1}}^{I} \sum_{i_{A2}\neq i_{A1}\neq i_{B1}}^{I} \sum_{i_{B2}=i_{A1}}^{I} \sum_{j_{A1}}^{J_{i_{A1}}} \sum_{j_{B1}}^{J_{i_{B1}}} \sum_{j_{A2}}^{J_{i_{A2}}} \sum_{j_{B2}}^{J_{i_{B2}}} \sum_{k_{A1}}^{K_{i_{A1}j_{A1}}} \sum_{k_{B1}}^{K_{i_{B1}j_{B1}}} \sum_{k_{A2}}^{K_{i_{A2}j_{A2}}} \sum_{k_{B2}}^{K_{i_{B2}j_{B2}}} (1-\delta_{i_{A1}j_{A1}k_{A1}})\delta_{i_{B1}j_{B1}k_{B1}}\times\\ (1-\delta_{i_{A2}j_{A2}k_{A2}})\delta_{i_{A1}j_{B2}k_{B2}}COV\left[I(S_{i_{A1}j_{A1}k_{A1}}-S_{i_{B1}j_{B1}k_{B1}}),I(S_{i_{A2}j_{A2}k_{A2}}-S_{i_{A1}j_{B2}k_{B2}})\right]= \end{matrix}$$

$$\begin{matrix} \sum_{i_{A1}=i_{B2}}^{I} \sum_{j_{A1}}^{J_{i_{A1}}} \sum_{j_{B1}}^{J_{i_{B1}}} \sum_{j_{A2}}^{J_{i_{A2}}} \sum_{j_{B2}\neq j_{A1}}^{J_{i_{B2}}} \sum_{k_{A1}}^{K_{i_{A1}j_{A1}}} \sum_{k_{B1}}^{K_{i_{B1}j_{B1}}} \sum_{k_{A2}}^{K_{i_{A2}j_{A2}}} \sum_{k_{B2}}^{K_{i_{B2}j_{B2}}} (1-\delta_{i_{A1}j_{A1}k_{A1}})\delta_{i_{B1}j_{B1}k_{B1}}(1-\delta_{i_{A2}j_{A2}k_{A2}})\delta_{i_{A1}j_{B2}k_{B2}} \\ COV\left[I(S_{i_{A1}j_{A1}k_{A1}}-S_{i_{B1}j_{B1}k_{B1}}),I(S_{i_{A2}j_{A2}k_{A2}}-S_{i_{A1}j_{B2}k_{B2}})\right]+ \end{matrix}$$

$$\left(C_{2,1}^{*}\right)$$

$$\begin{matrix} \sum_{i_{A1}=i_{B2}}^{I} \sum_{j_{A1}}^{J_{i_{A1}}} \sum_{j_{B1}}^{J_{i_{B1}}} \sum_{j_{A2}}^{J_{i_{A2}}} \sum_{j_{B2}=j_{A1}}^{J_{i_{B2}}} \sum_{k_{A1}}^{K_{i_{A1}j_{A1}}} \sum_{k_{B1}}^{K_{i_{B1}j_{B1}}} \sum_{k_{A2}}^{K_{i_{A2}j_{A2}}} \sum_{k_{B2}\neq k_{A1}}^{K_{i_{B2}j_{B2}}} (1-\delta_{i_{A1}j_{A1}k_{A1}})\delta_{i_{B1}j_{B1}k_{B1}}(1-\delta_{i_{A2}j_{A2}k_{A2}})\delta_{i_{A1}j_{A1}k_{B2}} \\ COV\left[I(S_{i_{A1}j_{A1}k_{A1}}-S_{i_{B1}j_{B1}k_{B1}}),I(S_{i_{A2}j_{A2}k_{A2}}-S_{i_{A1}j_{A1}k_{B2}})\right]+ \end{matrix}$$

Note that $k_{A1}=k_{B2}$does not contribute to the variance summation

$$\begin{matrix} C_{2,1}^{*}=\left[\Phi_{2}\left(\Phi^{-1}(\theta),\Phi^{-1}(\theta),\frac{-\rho_{f}}{2}\right)-\theta^{2}\right]\sum_{i_{A1}=i_{B2}}^{I} \sum_{j_{A1}}^{J_{i_{A1}}} \sum_{j_{B1}}^{J_{i_{B1}}} \sum_{j_{A2}}^{J_{i_{A2}}} \sum_{j_{B2}\neq j_{A1}}^{J_{i_{B2}}} \sum_{k_{A1}}^{K_{i_{A1}j_{A1}}} (1-\delta_{i_{A1}j_{A1}k_{A1}})\times\\ \sum_{k_{B1}}^{K_{i_{B1}j_{B1}}} \delta_{i_{B1}j_{B1}k_{B1}}\sum_{k_{A2}}^{K_{i_{A2}j_{A2}}} (1-\delta_{i_{A2}j_{A2}k_{A2}})\sum_{k_{B2}}^{K_{i_{B2}j_{B2}}} \delta_{i_{A1}j_{B2}k_{B2}}= \\ \left[\Phi_{2}\left(\Phi^{-1}(\theta),\Phi^{-1}(\theta),\frac{-\rho_{f}}{2}\right)-\theta^{2}\right]\sum_{i_{A1}=i_{B2}}^{I} \sum_{j_{B1}}^{J_{i_{B1}}} f_{i_{B1}j_{B1}}\sum_{j_{A2}}^{J_{i_{A2}}} g_{i_{A2}j_{A2}}\sum_{j_{A1}}^{J_{i_{A1}}} \sum_{j_{B2}\neq j_{A1}}^{J_{i_{B2}}} g_{i_{A1}j_{A1}}f_{i_{A1}j_{B2}}= \\ \left[\Phi_{2}\left(\Phi^{-1}(\theta),\Phi^{-1}(\theta),\frac{-\rho_{f}}{2}\right)-\theta^{2}\right]\sum_{i_{A1}=i_{B2}}^{I} f_{i_{B1}}g_{i_{A2}}\sum_{j_{A1}}^{J_{i_{A1}}} \sum_{j_{B2}\neq j_{A1}}^{J_{i_{B2}}} g_{i_{A1}j_{A1}}f_{i_{A1}j_{B2}}= \\ \left[\Phi_{2}\left(\Phi^{-1}(\theta),\Phi^{-1}(\theta),\frac{-\rho_{f}}{2}\right)-\theta^{2}\right]\sum_{i_{A1}=i_{B2}}^{I} f_{i_{B1}}g_{i_{A2}}\left(f_{i_{B2}}g_{i_{A1}}-\sum_{j_{A1}}^{J_{i_{A1}}} f_{i_{A1}j_{B2}}g_{i_{A1}j_{A1}}\right) \end{matrix}$$

$$\begin{matrix} C_{2,2}^{*}=\left[\Phi_{2}\left(\Phi^{-1}(\theta),\Phi^{-1}(\theta),\frac{-\rho_{s}}{2}\right)-\theta^{2}\right]\sum_{i_{A1}=i_{B2}}^{M} \sum_{j_{A1}}^{J_{i_{A1}}} \sum_{j_{B1}}^{J_{i_{B1}}} \sum_{j_{A2}}^{J_{i_{A2}}} \sum_{j_{B2}=j_{A1}}^{J_{i_{B2}}} \sum_{k_{A1}}^{K_{i_{A1}j_{A1}}} (1-\delta_{i_{A1}j_{A1}k_{A1}})\times\\ \sum_{k_{B1}}^{K_{i_{B1}j_{B1}}} \delta_{i_{B1}j_{B1}k_{B1}}\sum_{k_{A2}}^{K_{i_{A2}j_{A2}}} (1-\delta_{i_{A2}j_{A2}k_{A2}})\sum_{k_{B2}\neq k_{A1}}^{K_{i_{B2}j_{B2}}} \delta_{i_{A1}j_{A1}k_{B2}}g= \\ \left[\Phi_{2}\left(\Phi^{-1}(\theta),\Phi^{-1}(\theta),\frac{-\rho_{s}}{2}\right)-\theta^{2}\right]\sum_{i_{A1}=i_{B2}}^{M} f_{i_{B1}}g_{i_{A2}}\sum_{j_{A1}}^{J_{i_{A1}}} \sum_{k_{A1}}^{K_{i_{A1}j_{A1}}} (1-\delta_{i_{A1}j_{A1}k_{A1}})\sum_{k_{B2}\neq k_{A1}}^{K_{i_{B2}j_{B2}}} \delta_{i_{A1}j_{A1}k_{B2}}= \\ \left[\Phi_{2}\left(\Phi^{-1}(\theta),\Phi^{-1}(\theta),\frac{-\rho_{s}}{2}\right)-\theta^{2}\right]\sum_{i_{A1}=i_{B2}}^{M} f_{i_{B1}}g_{i_{A2}}\sum_{j_{A1}}^{J_{i_{A1}}} \sum_{k_{A1}}^{K_{i_{A1}j_{A1}}} (1-\delta_{i_{A1}j_{A1}k_{A1}})(f_{i_{A1}j_{A1}}-\delta_{i_{A1}j_{A1}k_{A1}})= \\ \left[\Phi_{2}\left(\Phi^{-1}(\theta),\Phi^{-1}(\theta),\frac{-\rho_{s}}{2}\right)-\theta^{2}\right]\sum_{i_{A1}=i_{B2}}^{M} f_{i_{B1}}g_{i_{A2}}\sum_{j_{A1}}^{J_{i_{A1}}} \sum_{k_{A1}}^{K_{i_{A1}j_{A1}}} f_{i_{A1}j_{A1}}-\delta_{i_{A1}j_{A1}k_{A1}}-\delta_{i_{A1}j_{A1}k_{A1}}f_{i_{A1}j_{A1}}+ \\ \delta_{i_{A1}j_{A1}k_{A1}}\delta_{i_{A1}j_{A1}k_{A1}}= \\ \left[\Phi_{2}\left(\Phi^{-1}(\theta),\Phi^{-1}(\theta),\frac{-\rho_{s}}{2}\right)-\theta^{2}\right]\sum_{i_{A1}=i_{B2}}^{M} f_{i_{B1}}g_{i_{A2}}\sum_{j_{A1}}^{J_{i_{A1}}} n_{i_{A1}j_{A1}}f_{i_{A1}j_{A1}}-f_{_{i_{A1}j_{A1}}}^{2} \end{matrix}$$

$C_{2}^{*}=C_{2,1}^{*}+C_{2,2}^{*}$ (13)

$$\begin{matrix} C_{3}^{*}=\sum_{i_{A1}}^{I} \sum_{i_{B1}\neq i_{A1}}^{I} \sum_{i_{A2}=i_{A1}}^{I} \sum_{i_{B2}\neq i_{A1}\neq i_{B1}}^{I} \sum_{j_{A1}}^{J_{i_{A1}}} \sum_{j_{B1}}^{J_{i_{B1}}} \sum_{j_{A2}}^{J_{i_{A2}}} \sum_{j_{B2}}^{J_{i_{B2}}} \sum_{k_{A1}}^{K_{i_{A1}j_{A1}}} \sum_{k_{B1}}^{K_{i_{B1}j_{B1}}} \sum_{k_{A2}}^{K_{i_{A2}j_{A2}}} \sum_{k_{B2}}^{K_{i_{B2}j_{B2}}} (1-\delta_{i_{A1}j_{A1}k_{A1}})\delta_{i_{B1}j_{B1}k_{B1}}\times\\ (1-\delta_{i_{A1}j_{A2}k_{A2}})\delta_{i_{B2}j_{B2}k_{B2}}COV\left[I(S_{i_{A1}j_{A1}k_{A1}}-S_{i_{B1}j_{B1}k_{B1}}),I(S_{i_{A1}j_{A2}k_{A2}}-S_{i_{B2}j_{B2}k_{B2}})\right]= \end{matrix}$$

$$\begin{matrix} \sum_{i_{A1}=i_{A2}}^{I} \sum_{j_{A1}}^{J_{i_{A1}}} \sum_{j_{B1}}^{J_{i_{B1}}} \sum_{j_{A2}\neq j_{A1}}^{J_{i_{A2}}} \sum_{j_{B2}}^{J_{i_{B2}}} \sum_{k_{A1}}^{K_{i_{A1}j_{A1}}} \sum_{k_{B1}}^{K_{i_{B1}j_{B1}}} \sum_{k_{A2}}^{K_{i_{A2}j_{A2}}} \sum_{k_{B2}}^{K_{i_{B2}j_{B2}}} (1-\delta_{i_{A1}j_{A1}k_{A1}})\delta_{i_{B1}j_{B1}k_{B1}}(1-\delta_{i_{A1}j_{A2}k_{A2}})\delta_{i_{B2}j_{B2}k_{B2}} \\ COV\left[I(S_{i_{A1}j_{A1}k_{A1}}-S_{i_{B1}j_{B1}k_{B1}}),I(S_{i_{A1}j_{A2}k_{A2}}-S_{i_{B2}j_{B2}k_{B2}})\right]+ \end{matrix}$$

$$\left(C_{3,1}^{*}\right)$$

$$\begin{matrix} \sum_{i_{A1}=i_{A2}}^{I} \sum_{j_{A1}}^{J_{i_{A1}}} \sum_{j_{B1}}^{J_{i_{B1}}} \sum_{j_{A2}=j_{A1}}^{J_{i_{A2}}} \sum_{j_{B2}}^{J_{i_{B2}}} \sum_{k_{A1}}^{K_{i_{A1}j_{A1}}} \sum_{k_{B1}}^{K_{i_{B1}j_{B1}}} \sum_{k_{A2}\neq k_{A1}}^{K_{i_{A2}j_{A2}}} \sum_{k_{B2}}^{K_{i_{B2}j_{B2}}} (1-\delta_{i_{A1}j_{A1}k_{A1}})\delta_{i_{B1}j_{B1}k_{B1}}(1-\delta_{i_{A1}j_{A1}k_{A2}})\delta_{i_{B2}j_{B2}k_{B2}} \\ COV\left[I(S_{i_{A1}j_{A1}k_{A1}}-S_{i_{B1}j_{B1}k_{B1}}),I(S_{i_{A1}j_{A1}k_{A2}}-S_{i_{B2}j_{B2}k_{B2}})\right]+ \end{matrix}$$

$$\left(C_{3,2}^{*}\right)$$

$$\begin{matrix} \sum_{i_{A1}=i_{A2}}^{I} \sum_{j_{A1}}^{J_{i_{A1}}} \sum_{j_{B1}}^{J_{i_{B1}}} \sum_{j_{A2}=j_{A1}}^{J_{i_{A2}}} \sum_{j_{B2}}^{J_{i_{B2}}} \sum_{k_{A1}}^{K_{i_{A1}j_{A1}}} \sum_{k_{B1}}^{K_{i_{B1}j_{B1}}} \sum_{k_{A2}=k_{A1}}^{K_{i_{A2}j_{A2}}} \sum_{k_{B2}}^{K_{i_{B2}j_{B2}}} (1-\delta_{i_{A1}j_{A1}k_{A1}})\delta_{i_{B1}j_{B1}k_{B1}}(1-\delta_{i_{A1}j_{A1}k_{A1}})\delta_{i_{B2}j_{B2}k_{B2}} \\ COV\left[I(S_{i_{A1}j_{A1}k_{A1}}-S_{i_{B1}j_{B1}k_{B1}}),I(S_{i_{A1}j_{A1}k_{A1}}-S_{i_{B2}j_{B2}k_{B2}})\right] \end{matrix}$$

$$\left(C_{3,3}^{*}\right)$$

$$\begin{matrix} C_{3,1}^{*}=\left[\Phi_{2}\left(\Phi^{-1}(\theta),\Phi^{-1}(\theta),\frac{\rho_{f}}{2}\right)-\theta^{2}\right]\sum_{i_{A1}=i_{A2}}^{I} \sum_{j_{A1}}^{J_{i_{A1}}} \sum_{j_{B1}}^{J_{i_{B1}}} \sum_{j_{A2}\neq j_{A1}}^{J_{i_{A2}}} \sum_{j_{B2}}^{J_{i_{B2}}} \sum_{k_{A1}}^{K_{i_{A1}j_{A1}}} (1-\delta_{i_{A1}j_{A1}k_{A1}})\times\\ \sum_{k_{B1}}^{K_{i_{B1}j_{B1}}} \delta_{i_{B1}j_{B1}k_{B1}}\sum_{k_{A2}}^{K_{i_{A2}j_{A2}}} (1-\delta_{i_{A1}j_{A2}k_{A2}})\sum_{k_{B2}}^{K_{i_{B2}j_{B2}}} \delta_{i_{B2}j_{B2}k_{B2}}= \\ \left[\Phi_{2}\left(\Phi^{-1}(\theta),\Phi^{-1}(\theta),\frac{\rho_{f}}{2}\right)-\theta^{2}\right]\sum_{i_{A1}=i_{A2}}^{I} \sum_{j_{A1}}^{J_{i_{A1}}} \sum_{j_{B1}}^{J_{i_{B1}}} \sum_{j_{A2}\neq j_{A1}}^{J_{i_{A2}}} \sum_{j_{B2}}^{J_{i_{B2}}} g_{i_{A1}j_{A1}}f_{i_{B1}j_{B1}}g_{i_{A2}j_{A2}}f_{i_{A1}j_{B2}}= \\ \left[\Phi_{2}\left(\Phi^{-1}(\theta),\Phi^{-1}(\theta),\frac{\rho_{f}}{2}\right)-\theta^{2}\right]\sum_{i_{A1}=i_{A2}}^{I} f_{i_{B1}}f_{i_{B2}}\left(g_{i_{A1}}^{2}-\sum_{j_{A1}}^{J_{i_{A1}}} g_{i_{A1}j_{A1}}^{2}\right) \end{matrix}$$

$$\begin{matrix} C_{3,2}^{*}=\left[\Phi_{2}\left(\Phi^{-1}(\theta),\Phi^{-1}(\theta),\frac{\rho_{s}}{2}\right)-\theta^{2}\right]\sum_{i_{A1}=i_{A2}}^{I} \sum_{j_{A1}}^{J_{i_{A1}}} \sum_{j_{B1}}^{J_{i_{B1}}} \sum_{j_{A2}=j_{A1}}^{J_{i_{A2}}} \sum_{j_{B2}}^{J_{i_{B2}}} \sum_{k_{A1}}^{K_{i_{A1}j_{A1}}} (1-\delta_{i_{A1}j_{A1}k_{A1}})\times\\ \sum_{k_{B1}}^{K_{i_{B1}j_{B1}}} \delta_{i_{B1}j_{B1}k_{B1}}\sum_{k_{A2}\neq k_{A1}}^{K_{i_{A2}j_{A2}}} (1-\delta_{i_{A1}j_{A1}k_{A2}})\sum_{k_{B2}}^{K_{i_{B2}j_{B2}}} \delta_{i_{B2}j_{B2}k_{B2}}= \\ \left[\Phi_{2}\left(\Phi^{-1}(\theta),\Phi^{-1}(\theta),\frac{\rho_{s}}{2}\right)-\theta^{2}\right]\sum_{i_{A1}=i_{A2}}^{I} f_{i_{B1}}f_{i_{B2}}\sum_{j_{A1}=j_{A2}}^{J_{i_{A1}}} \sum_{k_{A1}}^{K_{i_{A1}j_{A1}}} (1-\delta_{i_{A1}j_{A1}k_{A1}})\sum_{k_{A2}\neq k_{A1}}^{K_{i_{A2}j_{A2}}} (1-\delta_{i_{A1}j_{A1}k_{A2}}) \end{matrix}$$

$$\begin{matrix} C_{3,3}^{*}=\left[\Phi_{2}\left(\Phi^{-1}(\theta),\Phi^{-1}(\theta),\frac{1}{2}\right)-\theta^{2}\right]\sum_{i_{A1}=i_{A2}}^{I} \sum_{j_{A1}}^{J_{i_{A1}}} \sum_{j_{B1}}^{J_{i_{B1}}} \sum_{j_{A2}=j_{A1}}^{J_{i_{A2}}} \sum_{j_{B2}}^{J_{i_{B2}}} \sum_{k_{A1}}^{K_{i_{A1}j_{A1}}} (1-\delta_{i_{A1}j_{A1}k_{A1}})\times\\ \sum_{k_{B1}}^{K_{i_{B1}j_{B1}}} \delta_{i_{B1}j_{B1}k_{B1}}\sum_{k_{A2}=k_{A1}}^{K_{i_{A2}j_{A2}}} (1-\delta_{i_{A1}j_{A1}k_{A1}})\sum_{k_{B2}}^{K_{i_{B2}j_{B2}}} \delta_{i_{B2}j_{B2}k_{B2}}= \\ \left[\Phi_{2}\left(\Phi^{-1}(\theta),\Phi^{-1}(\theta),\frac{1}{2}\right)-\theta^{2}\right]\sum_{i_{A1}=i_{A2}}^{I} f_{i_{B1}}f_{i_{B2}}\sum_{j_{A2}=j_{A1}}^{J_{i_{A2}}} g_{i_{A1}j_{A1}}= \\ \left[\Phi_{2}\left(\Phi^{-1}(\theta),\Phi^{-1}(\theta),\frac{1}{2}\right)-\theta^{2}\right]\sum_{i_{A1}=i_{A2}}^{I} f_{i_{B1}}f_{i_{B2}}g_{i_{A1}} \end{matrix}$$

$C_{3}^{*}=C_{3,1}^{*}+C_{3,2}^{*}+C_{3,3}^{*}$ (14)

$$\begin{matrix} C_{4}^{*}=\sum_{i_{A1}}^{I} \sum_{i_{B1}\neq i_{A1}}^{I} \sum_{i_{A2}\neq i_{A1}}^{I} \sum_{i_{B2}=i_{B1}}^{I} \sum_{j_{A1}}^{J_{i_{A1}}} \sum_{j_{B1}}^{J_{i_{B1}}} \sum_{j_{A2}}^{J_{i_{A2}}} \sum_{j_{B2}}^{J_{i_{B2}}} \sum_{k_{A1}}^{K_{i_{A1}j_{A1}}} \sum_{k_{B1}}^{K_{i_{B1}j_{B1}}} \sum_{k_{A2}}^{K_{i_{A2}j_{A2}}} \sum_{k_{B2}}^{K_{i_{B2}j_{B2}}} (1-\delta_{i_{A1}j_{A1}k_{A1}})\delta_{i_{B1}j_{B1}k_{B1}}\times\\ (1-\delta_{i_{A2}j_{A2}k_{A2}})\delta_{i_{B1}j_{B2}k_{B2}}COV\left(I(S_{i_{A1}j_{A1}k_{A1}}-S_{i_{B1}j_{B1}k_{B1}}),I(S_{i_{A2}j_{A2}k_{A2}}-S_{i_{B1}j_{B2}k_{B2}})\right)= \end{matrix}$$

$$\begin{matrix} \sum_{i_{B1}=i_{B2}}^{I} \sum_{j_{A1}}^{J_{i_{A1}}} \sum_{j_{B1}}^{J_{i_{B1}}} \sum_{j_{A2}}^{J_{i_{A2}}} \sum_{j_{B2}\neq j_{B1}}^{J_{i_{B2}}} \sum_{k_{A1}}^{K_{i_{A1}j_{A1}}} \sum_{k_{B1}}^{K_{i_{B1}j_{B1}}} \sum_{k_{A2}}^{K_{i_{A2}j_{A2}}} \sum_{k_{B2}}^{K_{i_{B2}j_{B2}}} (1-\delta_{i_{A1}j_{A1}k_{A1}})\delta_{i_{B1}j_{B1}k_{B1}}(1-\delta_{i_{A2}j_{A2}k_{A2}})\delta_{i_{B1}j_{B2}k_{B2}} \\ COV\left[I(S_{i_{A1}j_{A1}k_{A1}}-S_{i_{B1}j_{B1}k_{B1}}),I(S_{i_{A2}j_{A2}k_{A2}}-S_{i_{B1}j_{B2}k_{B2}})\right]+ \end{matrix}$$

$$\left(C_{4,1}^{*}\right)$$

$$\begin{matrix} \sum_{i_{B1}=i_{B2}}^{I} \sum_{j_{A1}}^{J_{i_{A1}}} \sum_{j_{B1}}^{J_{i_{B1}}} \sum_{j_{A2}}^{J_{i_{A2}}} \sum_{j_{B2}=j_{B1}}^{J_{i_{B2}}} \sum_{k_{A1}}^{K_{i_{A1}j_{A1}}} \sum_{k_{B1}}^{K_{i_{B1}j_{B1}}} \sum_{k_{A2}}^{K_{i_{A2}j_{A2}}} \sum_{k_{B2}\neq k_{B1}}^{K_{i_{B2}j_{B2}}} (1-\delta_{i_{A1}j_{A1}k_{A1}})\delta_{i_{B1}j_{B1}k_{B1}}(1-\delta_{i_{A2}j_{A2}k_{A2}})\delta_{i_{B1}j_{B1}k_{B2}} \\ COV\left[I(S_{i_{A1}j_{A1}k_{A1}}-S_{i_{B1}j_{B1}k_{B1}}),I(S_{i_{A2}j_{A2}k_{A2}}-S_{i_{B1}j_{B1}k_{B2}})\right]+ \end{matrix}$$

$$\left(C_{4,2}^{*}\right)$$

$$\begin{matrix} \sum_{i_{A1}=i_{A2}}^{I} \sum_{j_{A1}}^{J_{i_{A1}}} \sum_{j_{B1}}^{J_{i_{B1}}} \sum_{j_{A2}}^{J_{i_{A2}}} \sum_{j_{B2}=j_{B1}}^{J_{i_{B2}}} \sum_{k_{A1}}^{K_{i_{A1}j_{A1}}} \sum_{k_{B1}}^{K_{i_{B1}j_{B1}}} \sum_{k_{A2}}^{K_{i_{A2}j_{A2}}} \sum_{k_{B2}=k_{B1}}^{K_{i_{B2}j_{B2}}} (1-\delta_{i_{A1}j_{A1}k_{A1}})\delta_{i_{B1}j_{B1}k_{B1}}(1-\delta_{i_{A2}j_{A2}k_{A2}})\delta_{i_{B1}j_{B1}k_{B1}} \\ COV\left[I(S_{i_{A1}j_{A1}k_{A1}}-S_{i_{B1}j_{B1}k_{B1}}),I(S_{i_{A2}j_{A2}k_{A2}}-S_{i_{B1}j_{B1}k_{B1}})\right] \end{matrix}$$

$$\left(C_{4,3}^{*}\right)$$

$$\begin{matrix} C_{4,1}^{*}=\left[\Phi_{2}\left(\Phi^{-1}(\theta),\Phi^{-1}(\theta),\frac{\rho_{f}}{2}\right)-\theta^{2}\right]\sum_{i_{B1}=i_{B2}}^{I} \sum_{j_{A1}}^{J_{i_{A1}}} \sum_{j_{B1}}^{J_{i_{B1}}} \sum_{j_{A2}}^{J_{i_{A2}}} \sum_{j_{B2}\neq j_{B1}}^{J_{i_{B2}}} \sum_{k_{A1}}^{K_{i_{A1}j_{A1}}} (1-\delta_{i_{A1}j_{A1}k_{A1}})\times\\ \sum_{k_{B1}}^{K_{i_{B1}j_{B1}}} \delta_{i_{B1}j_{B1}k_{B1}}\sum_{k_{A2}}^{K_{i_{A2}j_{A2}}} (1-\delta_{i_{A2}j_{A2}k_{A2}})\sum_{k_{B2}}^{K_{i_{B2}j_{B2}}} \delta_{i_{B1}j_{B2}k_{B2}}= \\ \left[\Phi_{2}\left(\Phi^{-1}(\theta),\Phi^{-1}(\theta),\frac{\rho_{f}}{2}\right)-\theta^{2}\right]\sum_{i_{B1}=i_{B2}}^{I} \sum_{j_{A1}}^{J_{i_{A1}}} \sum_{j_{B1}}^{J_{i_{B1}}} \sum_{j_{A2}}^{J_{i_{A2}}} \sum_{j_{B2}\neq j_{B1}}^{J_{i_{B2}}} g_{i_{A1}j_{A1}}f_{i_{B1}j_{B1}}g_{i_{A2}j_{A2}}f_{i_{B1}j_{B2}}= \\ \left[\Phi_{2}\left(\Phi^{-1}(\theta),\Phi^{-1}(\theta),\frac{\rho_{f}}{2}\right)-\theta^{2}\right]\sum_{i_{B1}=i_{B2}}^{I} g_{i_{A1}}g_{i_{A2}}\left(f_{i_{B1}}^{2}-\sum_{j_{B1}}^{J_{i_{B1}}} f_{_{i_{B1}j_{B1}}}^{2}\right) \end{matrix}$$

$$\begin{matrix} C_{4,2}^{*}=\left[\Phi_{2}\left(\Phi^{-1}(\theta),\Phi^{-1}(\theta),\frac{\rho_{s}}{2}\right)-\theta^{2}\right]\sum_{i_{B1}=i_{B2}}^{I} \sum_{j_{A1}}^{J_{i_{A1}}} \sum_{j_{B1}}^{J_{i_{B1}}} \sum_{j_{A2}}^{J_{i_{A2}}} \sum_{j_{B2}=j_{B1}}^{J_{i_{B2}}} \sum_{k_{A1}}^{K_{i_{A1}j_{A1}}} (1-\delta_{i_{A1}j_{A1}k_{A1}})\times\\ \sum_{k_{B1}}^{K_{i_{B1}j_{B1}}} \delta_{i_{B1}j_{B1}k_{B1}}\sum_{k_{A2}}^{K_{i_{A2}j_{A2}}} (1-\delta_{i_{A2}j_{A2}k_{A2}})\sum_{k_{B2}\neq k_{B1}}^{K_{i_{B2}j_{B2}}} \delta_{i_{B1}j_{B1}k_{B2}}= \\ \left[\Phi_{2}\left(\Phi^{-1}(\theta),\Phi^{-1}(\theta),\frac{\rho_{s}}{2}\right)-\theta^{2}\right]\sum_{i_{B1}=i_{B2}}^{I} g_{i_{A1}}g_{i_{A2}}\sum_{j_{B1}=j_{B2}}^{J_{i_{A1}}} \sum_{k_{B1}}^{K_{i_{A1}j_{A1}}} \delta_{i_{B1}j_{B1}k_{B1}}\sum_{k_{B2}\neq k_{B1}}^{K_{i_{A2}j_{A2}}} \delta_{i_{B1}j_{B1}k_{B2}}= \\ \left[\Phi_{2}\left(\Phi^{-1}(\theta),\Phi^{-1}(\theta),\frac{\rho_{s}}{2}\right)-\theta^{2}\right]\sum_{i_{B1}=i_{B2}}^{I} g_{i_{A1}}g_{i_{A2}}\sum_{j_{B1}=j_{B2}}^{J_{i_{A1}}} f_{i_{B1}j_{B1}}^{2}-f_{i_{B1}j_{B1}} \end{matrix}$$

$$\begin{matrix} C_{4,3}^{*}=\left[\Phi_{2}\left(\Phi^{-1}(\theta),\Phi^{-1}(\theta),\frac{1}{2}\right)-\theta^{2}\right]\sum_{i_{B1}=i_{B2}}^{I} \sum_{j_{A1}}^{J_{i_{A1}}} \sum_{j_{B1}}^{J_{i_{B1}}} \sum_{j_{A2}}^{J_{i_{A2}}} \sum_{j_{B2}=j_{B1}}^{J_{i_{B2}}} \sum_{k_{A1}}^{K_{i_{A1}j_{A1}}} (1-\delta_{i_{A1}j_{A1}k_{A1}})\times\\ \sum_{k_{B1}}^{K_{i_{B1}j_{B1}}} \delta_{i_{B1}j_{B1}k_{B1}}\sum_{k_{A2}}^{K_{i_{A2}j_{A2}}} (1-\delta_{i_{A2}j_{A2}k_{A2}})\sum_{k_{B2}=k_{B1}}^{K_{i_{B2}j_{B2}}} \delta_{i_{B1}j_{B1}k_{B1}}= \\ \left[\Phi_{2}\left(\Phi^{-1}(\theta),\Phi^{-1}(\theta),\frac{1}{2}\right)-\theta^{2}\right]\sum_{i_{B1}=i_{B2}}^{I} g_{i_{A1}}g_{i_{A2}}\sum_{j_{A2}=j_{A1}}^{J_{i_{A2}}} f_{i_{B1}j_{B1}}= \\ \left[\Phi_{2}\left(\Phi^{-1}(\theta),\Phi^{-1}(\theta),\frac{1}{2}\right)-\theta^{2}\right]\sum_{i_{B1}=i_{B2}}^{I} g_{i_{A1}}g_{i_{A2}}f_{i_{B1}} \end{matrix}$$

$C_{4}^{*}=C_{4,1}^{*}+C_{4,2}^{*}+C_{4,3}^{*}$ (15)

$$\begin{matrix} C_{5}^{*}=\sum_{i_{A1}}^{I} \sum_{i_{B1}\neq i_{A1}}^{I} \sum_{i_{A2}=i_{B1}}^{I} \sum_{i_{B2}\neq i_{B1}}^{I} \sum_{j_{A1}}^{J_{i_{A1}}} \sum_{j_{B1}}^{J_{i_{B1}}} \sum_{j_{A2}}^{J_{i_{A2}}} \sum_{j_{B2}}^{J_{i_{B2}}} \sum_{k_{A1}}^{K_{i_{A1}j_{A1}}} \sum_{k_{B1}}^{K_{i_{B1}j_{B1}}} \sum_{k_{A2}}^{K_{i_{A2}j_{A2}}} \sum_{k_{B2}}^{K_{i_{B2}j_{B2}}} (1-\delta_{i_{A1}j_{A1}k_{A1}})\delta_{i_{B1}j_{B1}k_{B1}}\times\\ (1-\delta_{i_{B1}j_{A2}k_{A2}})\delta_{i_{B2}j_{B2}k_{B2}}COV\left[I(S_{i_{A1}j_{A1}k_{A1}}-S_{i_{B1}j_{B1}k_{B1}}),I(S_{i_{B1}j_{A2}k_{A2}}-S_{i_{B2}j_{B2}k_{B2}})\right]= \end{matrix}$$

$$\begin{matrix} \sum_{i_{A2}=i_{B1}}^{I} \sum_{j_{A1}}^{J_{i_{A1}}} \sum_{j_{B1}}^{J_{i_{B1}}} \sum_{j_{A2}\neq j_{B1}}^{J_{i_{A2}}} \sum_{j_{B2}}^{J_{i_{B2}}} \sum_{k_{A1}}^{K_{i_{A1}j_{A1}}} \sum_{k_{B1}}^{K_{i_{B1}j_{B1}}} \sum_{k_{A2}}^{K_{i_{A2}j_{A2}}} \sum_{k_{B2}}^{K_{i_{B2}j_{B2}}} (1-\delta_{i_{A1}j_{A1}k_{A1}})\delta_{i_{B1}j_{B1}k_{B1}}(1-\delta_{i_{B1}j_{A2}k_{A2}})\delta_{i_{B2}j_{B2}k_{B2}} \\ COV\left[I(S_{i_{A1}j_{A1}k_{A1}}-S_{i_{B1}j_{B1}k_{B1}}),I(S_{i_{B1}j_{A2}k_{A2}}-S_{i_{B2}j_{B2}k_{B2}})\right]+ \end{matrix}$$

$$\left(C_{5,1}^{*}\right)$$

$$\begin{matrix} \sum_{i_{A2}=i_{B1}}^{I} \sum_{j_{A1}}^{J_{i_{A1}}} \sum_{j_{B1}}^{J_{i_{B1}}} \sum_{j_{A2}=j_{B1}}^{J_{i_{A2}}} \sum_{j_{B2}}^{J_{i_{B2}}} \sum_{k_{A1}}^{K_{i_{A1}j_{A1}}} \sum_{k_{B1}}^{K_{i_{B1}j_{B1}}} \sum_{k_{A2}\neq k_{B1}}^{K_{i_{A2}j_{A2}}} \sum_{k_{B2}}^{K_{i_{B2}j_{B2}}} (1-\delta_{i_{A1}j_{A1}k_{A1}})\delta_{i_{B1}j_{B1}k_{B1}}(1-\delta_{i_{B1}j_{B1}k_{A2}})\delta_{i_{B2}j_{B2}k_{B2}} \\ COV\left[I(S_{i_{A1}j_{A1}k_{A1}}-S_{i_{B1}j_{B1}k_{B1}}),I(S_{i_{B1}j_{B1}k_{A2}}-S_{i_{B2}j_{B2}k_{B2}})\right] \end{matrix}$$

$$\left(C_{5,2}^{*}\right)$$

Note that $k_{A2}=k_{B1}$does not contribute to the variance summation

$$\begin{matrix} C_{5,1}^{*}=\left[\Phi_{2}\left(\Phi^{-1}(\theta),\Phi^{-1}(\theta),\frac{-\rho_{f}}{2}\right)-\theta^{2}\right]\sum_{i_{A2}=i_{B1}}^{I} \sum_{j_{A1}}^{J_{i_{A1}}} \sum_{j_{B1}}^{J_{i_{B1}}} \sum_{j_{A2}\neq j_{B1}}^{J_{i_{A2}}} \sum_{j_{B2}}^{J_{i_{B2}}} \sum_{k_{A1}}^{K_{i_{A1}j_{A1}}} (1-\delta_{i_{A1}j_{A1}k_{A1}})\times\\ \sum_{k_{B1}}^{K_{i_{B1}j_{B1}}} \delta_{i_{B1}j_{B1}k_{B1}}\sum_{k_{A2}}^{K_{i_{A2}j_{A2}}} (1-\delta_{i_{B1}j_{A2}k_{A2}})\sum_{k_{B2}}^{K_{i_{B2}j_{B2}}} \delta_{i_{B2}j_{B2}k_{B2}}= \\ \left[\Phi_{2}\left(\Phi^{-1}(\theta),\Phi^{-1}(\theta),\frac{-\rho_{f}}{2}\right)-\theta^{2}\right]\sum_{i_{A2}=i_{B1}}^{I} \sum_{j_{A1}}^{J_{i_{A1}}} \sum_{j_{B1}}^{J_{i_{B1}}} \sum_{j_{A2}\neq j_{B1}}^{J_{i_{A2}}} \sum_{j_{B2}}^{J_{i_{B2}}} g_{i_{A1}j_{A1}}f_{i_{B1}j_{B1}}g_{i_{B1}j_{A2}}f_{i_{B2}j_{B2}}= \\ \left[\Phi_{2}\left(\Phi^{-1}(\theta),\Phi^{-1}(\theta),\frac{-\rho_{f}}{2}\right)-\theta^{2}\right]\sum_{i_{A2}=i_{B1}}^{I} g_{i_{A1}}f_{i_{B2}}\left(g_{i_{A2}}f_{i_{B1}}-\sum_{j_{B1}}^{J_{i_{B1}}} f_{i_{B1}j_{B1}}g_{i_{B1}j_{B1}}\right) \end{matrix}$$

$$\begin{matrix} C_{5,2}^{*}=\left[\Phi_{2}\left(\Phi^{-1}(\theta),\Phi^{-1}(\theta),\frac{-\rho_{s}}{2}\right)-\theta^{2}\right]\sum_{i_{A2}=i_{B1}}^{I} \sum_{j_{A1}}^{J_{i_{A1}}} \sum_{j_{B1}}^{J_{i_{B1}}} \sum_{j_{A2}=j_{B1}}^{J_{i_{A2}}} \sum_{j_{B2}}^{J_{i_{B2}}} \sum_{k_{A1}}^{K_{i_{A1}j_{A1}}} (1-\delta_{i_{A1}j_{A1}k_{A1}})\times\\ \sum_{k_{B1}}^{K_{i_{B1}j_{B1}}} \delta_{i_{B1}j_{B1}k_{B1}}\sum_{k_{A2}\neq k_{B1}}^{K_{i_{A2}j_{A2}}} (1-\delta_{i_{B1}j_{B1}k_{A2}})\sum_{k_{B2}}^{K_{i_{B2}j_{B2}}} \delta_{i_{B2}j_{B2}k_{B2}}= \\ \left[\Phi_{2}\left(\Phi^{-1}(\theta),\Phi^{-1}(\theta),\frac{-\rho_{s}}{2}\right)-\theta^{2}\right]\sum_{i_{A2}=i_{B1}}^{I} g_{i_{A1}}f_{i_{B2}}\sum_{j_{A2}=j_{B1}}^{J_{i_{A2}}} \sum_{k_{B1}}^{K_{i_{B1}j_{B1}}} \delta_{i_{B1}j_{B1}k_{B1}}\sum_{k_{A2}\neq k_{B1}}^{K_{i_{A2}j_{A2}}} (1-\delta_{i_{B1}j_{B1}k_{A2}})= \\ \left[\Phi_{2}\left(\Phi^{-1}(\theta),\Phi^{-1}(\theta),\frac{-\rho_{s}}{2}\right)-\theta^{2}\right]\sum_{i_{A2}=i_{B1}}^{I} g_{i_{A1}}f_{i_{B2}}\sum_{j_{A2}=j_{B1}}^{J_{i_{A2}}} n_{i_{B1}j_{B1}}f_{i_{B1}j_{B1}}-f^{2}_{i_{B1}j_{B1}} \end{matrix}$$

$C_{5}^{*}=C_{5,1}^{*}+C_{5,2}^{*}$ (16)

$$\begin{matrix} C_{6}^{*}=\sum_{i_{A1}}^{I} \sum_{i_{B1}\neq i_{A1}}^{I} \sum_{i_{A2}=i_{B1}}^{I} \sum_{i_{B2}=i_{A1}}^{I} \sum_{j_{A1}}^{J_{i_{A1}}} \sum_{j_{B1}}^{J_{i_{B1}}} \sum_{j_{A2}}^{J_{i_{A2}}} \sum_{j_{B2}}^{J_{i_{B2}}} \sum_{k_{A1}}^{K_{i_{A1}j_{A1}}} \sum_{k_{B1}}^{K_{i_{B1}j_{B1}}} \sum_{k_{A2}}^{K_{i_{A2}j_{A2}}} \sum_{k_{B2}}^{K_{i_{B2}j_{B2}}} (1-\delta_{i_{A1}j_{A1}k_{A1}})\delta_{i_{B1}j_{B1}k_{B1}}\times\\ (1-\delta_{i_{B1}j_{A2}k_{A2}})\delta_{i_{A1}j_{B2}k_{B2}}COV\left[I(S_{i_{A1}j_{A1}k_{A1}}-S_{i_{B1}j_{B1}k_{B1}}),I(S_{i_{B1}j_{A2}k_{A2}}-S_{i_{A1}j_{B2}k_{B2}})\right]= \end{matrix}$$

$$\begin{matrix} \sum_{i_{A2}=i_{B1},i_{B2}=i_{A1}}^{I} \sum_{j_{A1}}^{J_{i_{A1}}} \sum_{j_{B1}}^{J_{i_{B1}}} \sum_{j_{A2}\neq j_{B1}}^{J_{i_{A2}}} \sum_{j_{B2}\neq j_{A1}}^{J_{i_{B2}}} \sum_{k_{A1}}^{K_{i_{A1}j_{A1}}} \sum_{k_{B1}}^{K_{i_{B1}j_{B1}}} \sum_{k_{A2}}^{K_{i_{A2}j_{A2}}} \sum_{k_{B2}}^{K_{i_{B2}j_{B2}}} (1-\delta_{i_{A1}j_{A1}k_{A1}})\delta_{i_{B1}j_{B1}k_{B1}}(1-\delta_{i_{B1}j_{A2}k_{A2}})\delta_{i_{A1}j_{B2}k_{B2}} \\ COV\left[I(S_{i_{A1}j_{A1}k_{A1}}-S_{i_{B1}j_{B1}k_{B1}}),I(S_{i_{B1}j_{A2}k_{A2}}-S_{i_{A1}j_{B2}k_{B2}})\right]+ \end{matrix}$$

$$\left(C_{6,1}^{*}\right)$$

$$\begin{matrix} \sum_{i_{A2}=i_{B1},i_{B2}=i_{A1}}^{I} \sum_{j_{A1}}^{J_{i_{A1}}} \sum_{j_{B1}}^{J_{i_{B1}}} \sum_{j_{A2}\neq j_{B1}}^{J_{i_{A2}}} \sum_{j_{B2}=j_{A1}}^{J_{i_{B2}}} \sum_{k_{A1}}^{K_{i_{A1}j_{A1}}} \sum_{k_{B1}}^{K_{i_{B1}j_{B1}}} \sum_{k_{A2}}^{K_{i_{A2}j_{A2}}} \sum_{k_{B2}\neq k_{A1}}^{K_{i_{B2}j_{B2}}} (1-\delta_{i_{A1}j_{A1}k_{A1}})\delta_{i_{B1}j_{B1}k_{B1}}(1-\delta_{i_{B1}j_{A2}k_{A2}})\delta_{i_{A1}j_{A1}k_{B2}} \\ COV\left[I(S_{i_{A1}j_{A1}k_{A1}}-S_{i_{B1}j_{B1}k_{B1}}),I(S_{i_{B1}j_{A2}k_{A2}}-S_{i_{A1}j_{A1}k_{B2}})\right]+ \end{matrix}$$

$$\left(C_{6,2}^{*}\right)$$

$$\begin{matrix} \sum_{i_{A2}=i_{B1},i_{B2}=i_{A1}}^{I} \sum_{j_{A1}}^{J_{i_{A1}}} \sum_{j_{B1}}^{J_{i_{B1}}} \sum_{j_{A2}=j_{B1}}^{J_{i_{A2}}} \sum_{j_{B2}\neq j_{A1}}^{J_{i_{B2}}} \sum_{k_{A1}}^{K_{i_{A1}j_{A1}}} \sum_{k_{B1}}^{K_{i_{B1}j_{B1}}} \sum_{k_{A2}\neq k_{B1}}^{K_{i_{A2}j_{A2}}} \sum_{k_{B2}}^{K_{i_{B2}j_{B2}}} (1-\delta_{i_{A1}j_{A1}k_{A1}})\delta_{i_{B1}j_{B1}k_{B1}}(1-\delta_{i_{B1}j_{B1}k_{A2}})\delta_{i_{A1}j_{B2}k_{B2}} \\ COV\left[I(S_{i_{A1}j_{A1}k_{A1}}-S_{i_{B1}j_{B1}k_{B1}}),I(S_{i_{B1}j_{B1}k_{A2}}-S_{i_{A1}j_{B2}k_{B2}})\right]+ \end{matrix}$$

$$\left(C_{6,3}^{*}\right)$$

$$\begin{matrix} \sum_{i_{A2}=i_{B1},i_{B2}=i_{A1}}^{I} \sum_{j_{A1}}^{J_{i_{A1}}} \sum_{j_{B1}}^{J_{i_{B1}}} \sum_{j_{A2}=j_{B1}}^{J_{i_{A2}}} \sum_{j_{B2}=j_{A1}}^{J_{i_{B2}}} \sum_{k_{A1}}^{K_{i_{A1}j_{A1}}} \sum_{k_{B1}}^{K_{i_{B1}j_{B1}}} \sum_{k_{A2}\neq k_{B1}}^{K_{i_{A2}j_{A2}}} \sum_{k_{B2}\neq k_{A1}}^{K_{i_{B2}j_{B2}}} (1-\delta_{i_{A1}j_{A1}k_{A1}})\delta_{i_{B1}j_{B1}k_{B1}}(1-\delta_{i_{B1}j_{B1}k_{A2}})\delta_{i_{A1}j_{A1}k_{B2}} \\ COV\left[I(S_{i_{A1}j_{A1}k_{A1}}-S_{i_{B1}j_{B1}k_{B1}}),I(S_{i_{B1}j_{B1}k_{A2}}-S_{i_{A1}j_{A1}k_{B2}})\right]+ \end{matrix}$$

$$\left(C_{6,4}^{*}\right)$$

$$\begin{matrix} C_{6,1}^{*}=\left[\Phi_{2}\left(\Phi^{-1}(\theta),\Phi^{-1}(\theta),-\rho_{f}\right)-\theta^{2}\right]\sum_{i_{A2}=i_{B1},i_{B2}=i_{A1}}^{I} \sum_{j_{A1}}^{J_{i_{A1}}} \sum_{j_{B1}}^{J_{i_{B1}}} \sum_{j_{A2}\neq j_{B1}}^{J_{i_{A2}}} \sum_{j_{B2}\neq j_{A1}}^{J_{i_{B2}}} \sum_{k_{A1}}^{K_{i_{A1}j_{A1}}} (1-\delta_{i_{A1}j_{A1}k_{A1}})\times\\ \sum_{k_{B1}}^{K_{i_{B1}j_{B1}}} \delta_{i_{B1}j_{B1}k_{B1}}\sum_{k_{A2}}^{K_{i_{A2}j_{A2}}} (1-\delta_{i_{B1}j_{A2}k_{A2}})\sum_{k_{B2}}^{K_{i_{B2}j_{B2}}} \delta_{i_{A1}j_{B2}k_{B2}}= \\ \left[\Phi_{2}\left(\Phi^{-1}(\theta),\Phi^{-1}(\theta),-\rho_{f}\right)-\theta^{2}\right]\sum_{i_{A2}=i_{B1},i_{B2}=i_{A1}}^{I} \sum_{j_{A1}}^{J_{i_{A1}}} \sum_{j_{B1}}^{J_{i_{B1}}} \sum_{j_{A2}\neq j_{B1}}^{J_{i_{A2}}} \sum_{j_{B2}\neq j_{A1}}^{J_{i_{B2}}} g_{i_{A1}j_{A1}}f_{i_{B1}j_{B1}}g_{i_{B1}j_{A2}}f_{i_{A1}j_{B2}}= \\ \left[\Phi_{2}\left(\Phi^{-1}(\theta),\Phi^{-1}(\theta),-\rho_{f}\right)-\theta^{2}\right]\sum_{i_{A2}=i_{B1},i_{B2}=i_{A1}}^{I} \left(g_{i_{A2}}f_{i_{B1}}-\sum_{j_{B1}}^{J_{i_{B1}}} f_{i_{B1}j_{B1}}g_{i_{B1}j_{B1}}\right)\times\\ \left(g_{i_{A1}}f_{i_{B2}}-\sum_{j_{A1}}^{J_{i_{A1}}} f_{i_{A1}j_{A1}}g_{i_{A1}j_{A1}}\right) \end{matrix}$$

$$\begin{matrix} C_{6,3}^{*}=\left[\Phi_{2}\left(\Phi^{-1}(\theta),\Phi^{-1}(\theta),\frac{-\rho_{f}-\rho_{s}}{2}\right)-\theta^{2}\right]\sum_{i_{A2}=i_{B1},i_{B2}=i_{A1}}^{I} \sum_{j_{A1}}^{J_{i_{A1}}} \sum_{j_{B1}}^{J_{i_{B1}}} \sum_{j_{A2}=j_{B1}}^{J_{i_{A2}}} \sum_{j_{B2}\neq j_{A1}}^{J_{i_{B2}}} \sum_{k_{A1}}^{K_{i_{A1}j_{A1}}} (1-\delta_{i_{A1}j_{A1}k_{A1}})\times\\ \sum_{k_{B1}}^{K_{i_{B1}j_{B1}}} \delta_{i_{B1}j_{B1}k_{B1}}\sum_{k_{A2}\neq k_{B1}}^{K_{i_{A2}j_{A2}}} (1-\delta_{i_{B1}j_{B1}k_{A2}})\sum_{k_{B2}}^{K_{i_{B2}j_{B2}}} \delta_{i_{A1}j_{B2}k_{B2}}= \\ \left[\Phi_{2}\left(\Phi^{-1}(\theta),\Phi^{-1}(\theta),\frac{-\rho_{f}-\rho_{s}}{2}\right)-\theta^{2}\right]\sum_{i_{A2}=i_{B1},i_{B2}=i_{A1}}^{I} \sum_{j_{A1}}^{J_{i_{A1}}} \sum_{k_{A1}}^{K_{i_{A1}j_{A1}}} (1-\delta_{i_{A1}j_{A1}k_{A1}})\sum_{j_{B2}\neq j_{A1}}^{J_{i_{B2}}} \sum_{k_{B2}}^{K_{i_{B2}j_{B2}}} \delta_{i_{A1}j_{B2}k_{B2}}\times\\ \sum_{j_{A2}=j_{B1}}^{J_{i_{A2}}} \sum_{k_{B1}}^{K_{i_{B1}j_{B1}}} \delta_{i_{B1}j_{B1}k_{B1}}\sum_{k_{A2}\neq k_{B1}}^{K_{i_{A2}j_{A2}}} (1-\delta_{i_{B1}j_{B1}k_{A2}})= \\ \left[\Phi_{2}\left(\Phi^{-1}(\theta),\Phi^{-1}(\theta),\frac{-\rho_{f}-\rho_{s}}{2}\right)-\theta^{2}\right]\sum_{i_{A2}=i_{B1},i_{B2}=i_{A1}}^{I} (f_{i_{B2}}g_{i_{A1}}-\sum_{j_{A1}}^{J_{i_{A1}}} g_{i_{A1}j_{A1}}f_{i_{A1}j_{A1}})\sum_{j_{B1}}^{J_{i_{A2}}} n_{i_{B1}j_{B1}}f_{i_{B1}j_{B1}}-f^{2}_{i_{B1}j_{B1}} \end{matrix}$$

$$\begin{matrix} C_{6,4}^{*}=\left[\Phi_{2}\left(\Phi^{-1}(\theta),\Phi^{-1}(\theta),-\rho_{s}\right)-\theta^{2}\right]\sum_{i_{A2}=i_{B1},i_{B2}=i_{A1}}^{I} \sum_{j_{A1}}^{J_{i_{A1}}} \sum_{j_{B1}}^{J_{i_{B1}}} \sum_{j_{A2}=j_{B1}}^{J_{i_{A2}}} \sum_{j_{B2}=j_{A1}}^{J_{i_{B2}}} \sum_{k_{A1}}^{K_{i_{A1}j_{A1}}} (1-\delta_{i_{A1}j_{A1}k_{A1}})\times\\ \sum_{k_{B1}}^{K_{i_{B1}j_{B1}}} \delta_{i_{B1}j_{B1}k_{B1}}\sum_{k_{A2}\neq k_{B1}}^{K_{i_{A2}j_{A2}}} (1-\delta_{i_{B1}j_{B1}k_{A2}})\sum_{k_{B2}\neq k_{A1}}^{K_{i_{B2}j_{B2}}} \delta_{i_{A1}j_{A1}k_{B2}}= \\ \left[\Phi_{2}\left(\Phi^{-1}(\theta),\Phi^{-1}(\theta),-\rho_{s}\right)-\theta^{2}\right]\sum_{i_{A2}=i_{B1},i_{B2}=i_{A1}}^{I} \sum_{j_{A2}=j_{B1}}^{J_{i_{A2}}} \sum_{k_{B1}}^{K_{i_{B1}j_{B1}}} \delta_{i_{B1}j_{B1}k_{B1}}\sum_{k_{A2}\neq k_{B1}}^{K_{i_{A2}j_{A2}}} (1-\delta_{i_{B1}j_{B1}k_{A2}})\times\\ \sum_{j_{B2}=j_{A1}}^{J_{i_{B2}}} \sum_{k_{A1}}^{K_{i_{A1}j_{A1}}} (1-\delta_{i_{A1}j_{A1}k_{A1}})\sum_{k_{B2}\neq k_{A1}}^{K_{i_{B2}j_{B2}}} \delta_{i_{A1}j_{A1}k_{B2}}= \\ \left[\Phi_{2}\left(\Phi^{-1}(\theta),\Phi^{-1}(\theta),-\rho_{s}\right)-\theta^{2}\right]\sum_{i_{A2}=i_{B1},i_{B2}=i_{A1}}^{I} \sum_{j_{B1}}^{J_{i_{B1}}} \left(n_{i_{B1}j_{B1}}f_{i_{B1}j_{B1}}-f^{2}_{i_{B1}j_{B1}}\right)\sum_{j_{A1}}^{J_{i_{A1}}} \left(n_{i_{A1}j_{A1}}f_{i_{A1}j_{A1}}-f^{2}_{i_{A1}j_{A1}}\right) \end{matrix}$$

$C_{6}^{*}=C_{6,1}^{*}+C_{6,2}^{*}+C_{6,3}^{*}+C_{6,4}^{*}$ (17)

Thus,

$VAR(C)=C_{1}^{*}+C_{2}^{*}+C_{3}^{*}+C_{4}^{*}+C_{5}^{*}+C_{6}^{*}$ (18)

4. *COV(A,B)*

$$\begin{matrix} A=\sum_{i=1}^{I} \sum_{j=1}^{J_{i}} \sum_{k_{1}=1}^{K_{ij}} \sum_{k_{2}=1}^{K_{ij}} (1-\delta_{ijk_{1}})\delta_{ijk_{2}}I(S_{ijk_{1}}-S_{ijk_{2}}) \\ B=\sum_{i=1}^{I} \sum_{j_{1}=1}^{J_{i}} \sum_{j_{2}\neq j_{1}}^{J_{i}} \sum_{k_{1}=1}^{K_{ij_{1}}} \sum_{k_{2}=1}^{K_{ij_{2}}} (1-\delta_{ij_{1}k_{1}})\delta_{ij_{2}k_{2}}I(S_{ij_{1}k_{1}}-S_{ij_{2}k_{2}}) \\ COV(A,B)=COV\left[\begin{matrix} \sum_{i=1}^{I} \sum_{j=1}^{J_{i}} \sum_{k_{1}=1}^{K_{ij}} \sum_{k_{2}=1}^{K_{ij}} (1-\delta_{ijk_{1}})\delta_{ijk_{2}}I(S_{ijk_{1}}-S_{ijk_{2}}), \\ \sum_{i=1}^{I} \sum_{j_{1}=1}^{J_{i}} \sum_{j_{2}\neq j_{1}}^{J_{i}} \sum_{k_{1}=1}^{K_{ij_{1}}} \sum_{k_{2}=1}^{K_{ij_{2}}} (1-\delta_{ij_{1}k_{1}})\delta_{ij_{2}k_{2}}I(S_{ij_{1}k_{1}}-S_{ij_{2}k_{2}}) \end{matrix}\right]= \end{matrix}$$

(19)

There are five possible relatedness patterns in the covariance term. The family must be the same across A and B or the covariance is zero; additionally,or leads to a zero summation. Different indices imply inequality.

| *A* | *B* |  |
| --- | --- | --- |
| j_1_,k_1_ - j_1_,k_2_ | j_2_,k - j_3_,k | $AB_{1}^{*}$ |
| j_1_,k_1_ - j_1_,k_2_ | j_1_,k_1_ - j_2_,k | $AB_{2}^{*}$ |
| j_1_,k_1_ - j_1_,k_2_ | j_1_,k_3_ - j_2_,k | $AB_{3}^{*}$ |
| j_1_,k_1_ - j_1_,k_2_ | j_2_,k - j_1_,k_2_ | $AB_{4}^{*}$ |
| j_1_,k_1_ - j_1_,k_2_ | j_2_,k- j_1_,k_3_ | $AB_{5}^{*}$ |

Thus,

$\begin{matrix} COV(A,B)=\left[\Phi_{2}\left(\Phi^{-1}(\theta_{s}),\Phi^{-1}(\theta_{f}),\frac{(1-\rho_{s})}{\sqrt{2(1-\rho_{s})}\sqrt{2(1-\rho_{f})}}\right)-\theta_{s}\theta_{f}\right]\times\\ \sum_{i}^{I} \sum_{j_{A}}^{J_{i}} f_{ij_{A}}g_{ij_{A}}(f_{i}+g_{i}-f_{ij_{A}}-g_{ij_{A}}) \end{matrix}$ (20)

5. *COV(A,C)*

$$\begin{matrix} A=\sum_{i}^{I} \sum_{j}^{J_{i}} \sum_{k_{1}}^{K_{ij}} \sum_{k_{2}}^{K_{ij}} (1-\delta_{ijk_{1}})\delta_{ijk_{2}}I(S_{ijk_{1}}-S_{ijk_{2}}) \\ C=\sum_{i_{1}}^{I} \sum_{i_{2}\neq i_{1}}^{I} \sum_{j_{1}}^{J_{i_{1}}} \sum_{j_{2}}^{J_{i_{2}}} \sum_{k_{1}}^{K_{i_{1}j_{1}}} \sum_{k_{2}}^{K_{i_{2}j_{2}}} (1-\delta_{i_{1}j_{1}k_{1}})\delta_{i_{2}j_{2}k_{2}}I(S_{i_{1}j_{1}k_{1}}-S_{i_{2}j_{2}k_{2}}) \end{matrix}$$

(21)

There are six possible relatedness patterns in the covariance term.$S_{i_{A}j_{A}k_{A2}}=S_{i_{C1}j_{C1}k_{C1}}$or leads to a zero summation. Different indices imply inequality.

| *A* | *C* |  |
| --- | --- | --- |
| i_1_,j_1_,k_1_ - i_1_,j_1_,k_2_ | i_2_,j,k- i_3_,j,k | 0 |
| i_1_,j_1_,k_1_ - i_1_,j_1_,k_2_ | i_1_,j_1_,k_1_ - i_2_,j,k | $AC_{1}^{*}$ |
| i_1_,j_1_,k_1_ - i_1_,j_1_,k_2_ | i_1_,j_1_,k_3_ - i_3_,j,k | $AC_{2}^{*}$ |
| i_1_,j_1_,k_1_ - i_1_,j_1_,k_2_ | i_1_,j_2_,k- i_3_,j,k | $AC_{3}^{*}$ |
| i_1_,j_1_,k_1_ - i_1_,j_1_,k_2_ | i_2_,j,k- i_1_,j_1_,k_2_ | $AC_{4}^{*}$ |
| i_1_,j_1_,k_1_ - i_1_,j_1_,k_2_ | i_2_,j,k- i_1_,j_1_,k_3_ | $AC_{5}^{*}$ |
| i_1_,j_1_,k_1_ - i_1_,j_1_,k_2_ | i_2_,j,k- i_1_,j_2_,k | $AC_{6}^{*}$ |

Thus, $COV(A,C)=\left[\Phi_{2}\left(\Phi^{-1}(\theta_{s}),\Phi^{-1}(\theta),\frac{(1-\rho_{s})}{2\sqrt{(1-\rho_{s})}}\right)-\theta_{s}\theta\right]\sum_{i_{A}}^{I} \underline{fg}_{i_{A}}\left(N-n_{i_{A}}\right)$ (22)

6. *COV(B,C)*

$$\begin{matrix} B=\sum_{i}^{I} \sum_{j_{1}}^{J_{i}} \sum_{j_{2}\neq j_{1}}^{J_{i}} \sum_{k_{1}}^{K_{ij_{1}}} \sum_{k_{2}}^{K_{ij_{2}}} (1-\delta_{ij_{1}k_{1}})\delta_{ij_{2}k_{2}}I(S_{ij_{1}k_{1}}-S_{ij_{2}k_{2}}) \\ C=\sum_{i_{1}}^{I} \sum_{i_{2}\neq i_{1}}^{I} \sum_{j_{1}}^{J_{i_{1}}} \sum_{j_{2}}^{J_{i_{2}}} \sum_{k_{1}}^{K_{i_{1}j_{1}}} \sum_{k_{2}}^{K_{i_{2}j_{2}}} (1-\delta_{i_{1}j_{1}k_{1}})\delta_{i_{2}j_{2}k_{2}}I(S_{i_{1}j_{1}k_{1}}-S_{i_{2}j_{2}k_{2}}) \end{matrix}$$

$\begin{matrix} COV(B,C)=COV\left(\begin{matrix} \sum_{i}^{I} \sum_{j_{1}}^{J_{i}} \sum_{j_{2}\neq j_{1}}^{J_{i}} \sum_{k_{1}}^{K_{ij_{1}}} \sum_{k_{2}}^{K_{ij_{2}}} (1-\delta_{ij_{1}k_{1}})\delta_{ij_{2}k_{2}}I(S_{ij_{1}k_{1}}-S_{ij_{2}k_{2}}), \\ \sum_{i_{1}}^{I} \sum_{i_{2}\neq i_{1}}^{I} \sum_{j_{1}}^{J_{i_{1}}} \sum_{j_{2}}^{J_{i_{2}}} \sum_{k_{1}}^{K_{i_{1}j_{1}}} \sum_{k_{2}}^{K_{i_{2}j_{2}}} (1-\delta_{i_{1}j_{1}k_{1}})\delta_{i_{2}j_{2}k_{2}}I(S_{i_{1}j_{1}k_{1}}-S_{i_{2}j_{2}k_{2}}) \end{matrix}\right)= \\ \sum_{i_{B}}^{I} \sum_{j_{B1}}^{J_{i_{B}}} \sum_{j_{B2}\neq j_{B1}}^{J_{i_{B}}} \sum_{k_{B1}}^{K_{i_{B}j_{B1}}} \sum_{k_{B2}}^{K_{i_{B}j_{B2}}} \sum_{i_{C1}}^{I} \sum_{i_{C2}\neq i_{C1}}^{I} \sum_{j_{C1}}^{J_{i_{C1}}} \sum_{j_{C2}}^{J_{i_{C2}}} \sum_{k_{C1}}^{K_{i_{C1}j_{C1}}} \sum_{k_{C2}}^{K_{i_{C2}j_{C2}}} (1-\delta_{i_{B}j_{B1}k_{B1}})\delta_{i_{B}j_{B2}k_{B2}}(1-\delta_{i_{C1}j_{C1}k_{C1}})\delta_{i_{C2}j_{C2}k_{C2}} \\ COV(I(S_{i_{B}j_{B1}k_{B1}}-S_{i_{B}j_{B2}k_{B2}}),I(S_{i_{C1}j_{C1}k_{C1}}-S_{i_{C2}j_{C2}k_{C2}})) \end{matrix}$ (23)

There are eight possible relatedness patterns in the covariance term. $S_{i_{B}j_{B2}k_{B2}}=S_{i_{C1}j_{C1}k_{C1}}$ or $S_{i_{B}j_{B1}k_{B1}}=S_{i_{C2}j_{C2}k_{C2}}$ leads to a zero summation. Different indices imply inequality.

| *B* | *C* |  |
| --- | --- | --- |
| i_1_,j_1_,k_1_ - i_1_,j_2_,k_2_ | i_2_,j,k- i_3_,j,k | 0 |
| i_1_,j_1_,k_1_ - i_1_,j_2_,k_2_ | i_1_,j_1_,k_1_ - i_2_,j,k | $BC_{1}^{*}$ |
| i_1_,j_1_,k_1_ - i_1_,j_2_,k_2_ | i_1_,j_1_,k_3_ - i_2_,j,k | $BC_{2}^{*}$ |
| i_1_,j_1_,k_1_ - i_1_,j_2_,k_2_ | i_1_,j_2_,k_3_ - i_2_,j,k | $BC_{3}^{*}$ |
| i_1_,j_1_,k_1_ - i_1_,j_2_,k_2_ | i_1_,j_3_,k- i_2_,j,k | $BC_{4}^{*}$ |
| i_1_,j_1_,k_1_ - i_1_,j_2_,k_2_ | i_2_,j,k- i_1_,j_2_,k_2_ | $BC_{5}^{*}$ |
| i_1_,j_1_,k_1_ - i_1_,j_2_,k_2_ | i_2_,j,k- i_1_,j_2_,k_3_ | $BC_{6}^{*}$ |
| i_1_,j_1_,k_1_ - i_1_,j_2_,k_2_ | i_2_,j,k- i_1_,j_1_,k_3_ | $BC_{7}^{*}$ |
| i_1_,j_1_,k_1_ - i_1_,j_2_,k_2_ | i_2_,j,k- i_1_,j_3_,k | $BC_{8}^{*}$ |

$$\begin{matrix} BC_{1}^{*}=\sum_{i_{B},i_{C1}=i_{B}}^{I} \sum_{j_{B1},j_{C1}=j_{B1}}^{J_{i_{B}}} \sum_{j_{B2}\neq j_{B1}}^{J_{i_{B}}} \sum_{k_{B1},k_{C1}=k_{B1}}^{K_{i_{B}j_{B1}}} \sum_{k_{B2}}^{K_{i_{B}j_{B2}}} \sum_{i_{C2}\neq i_{C1}}^{I} \sum_{j_{C2}}^{J_{i_{C2}}} \sum_{k_{C2}}^{K_{i_{C2}j_{C2}}} (1-\delta_{i_{B}j_{B1}k_{B1}})\delta_{i_{B}j_{B2}k_{B2}}(1-\delta_{i_{B}j_{B1}k_{B1}})\delta_{i_{C2}j_{C2}k_{C2}} \\ COV\left[I(S_{i_{B}j_{B1}k_{B1}}-S_{i_{B}j_{B2}k_{B2}}),I(S_{i_{B}j_{B1}k_{B1}}-S_{i_{C2}j_{C2}k_{C2}})\right]= \\ \left[\Phi_{2}\left(\Phi^{-1}(\theta_{f}),\Phi^{-1}(\theta),\frac{1-\rho_{f}}{2\sqrt{(1-\rho_{f})}}\right)-\theta_{f}\theta\right]\sum_{i_{B},i_{C1}=i_{B}}^{I} \sum_{j_{B1},j_{C1}=j_{B1}}^{J_{i_{B}}} \sum_{k_{B1},k_{C1}=k_{B1}}^{K_{i_{B}j_{B1}}} (1-\delta_{i_{B}j_{B1}k_{B1}})\sum_{j_{B2}\neq j_{B1}}^{J_{i_{B}}} \sum_{k_{B2}}^{K_{i_{B}j_{B2}}} \delta_{i_{B}j_{B2}k_{B2}}\times\\ \sum_{i_{C2}\neq i_{C1}}^{I} \sum_{j_{C2}}^{J_{i_{C2}}} \sum_{k_{C2}}^{K_{i_{C2}j_{C2}}} \delta_{i_{C2}j_{C2}k_{C2}}= \\ \left[\Phi_{2}\left(\Phi^{-1}(\theta_{f}),\Phi^{-1}(\theta),\frac{1-\rho_{f}}{2\sqrt{(1-\rho_{f})}}\right)-\theta_{f}\theta\right]\sum_{i_{B},i_{C1}=i_{B}}^{I} (F-f_{i_{C1}})\sum_{j_{B1},j_{C1}=j_{B1}}^{J_{i_{B}}} g_{i_{B}j_{B1}}(f_{i_{B}}-f_{i_{B}j_{B1}}) \end{matrix}$$

Thus, $COV(B,C)=$

(24)

7. *VAR($\hat{\theta_{3}}$)*

$VAR(\hat{\theta_{3}})=\frac{1}{F^{2}\times G^{2}}\left[ VAR(A)+VAR(B)+VAR(C)+2COV(A,B)+2COV(A,C)+2COV(B,C) \right]$ (25)
